# Supplementary material for: Bayesian log-normal deconvolution for enhanced in silico microdissection of bulk gene expression data
Source: Nat Commun. 2021 Oct 20;12:6106. doi: 10.1038/s41467-021-26328-2 (PMC8528834; doi:10.1038/s41467-021-26328-2)
Supplement: Supplementary file 1 — Supplementary Information [file 41467_2021_26328_MOESM1_ESM.pdf]

Supplementary Information for:

Bayesian log-normal deconvolution for enhanced *in silico* microdissection of bulk gene expression data

Bárbara Andrade Barbosa[1], Saskia D. van Asten[1,2], Ji Won Oh[3,4], Arantza Farina Sarasqueta[5], Joanne Verheij[5], Frederike Dijk[5], Hanneke van Laarhoven[6], Bauke Ylstra[1], Juan Garcia Vallejo[2], Mark A. van de Wiel[7*], Yongsoo Kim[1*]

[1]Department of Pathology, Cancer-Center Amsterdam, Amsterdam UMC location VUmc, 1081 HV, Amsterdam, the Netherlands

[2]Department of Molecular Cell Biology & Immunology, Amsterdam UMC location VUmc, Amsterdam Infection and immunity Institute, 1081 HZ, Amsterdam, the Netherlands

[3]Department of Anatomy, School of Medicine, Kyungpook National University, 41940, Daegu, South Korea

[4]Bio-Medical Research Institute, Kyungpook National University Hospital, 41944, Daegu, South Korea

[5]Department of Pathology, Amsterdam UMC location AMC, 1105 AZ, Amsterdam, the Netherlands

[6]Department of Medical Oncology, Cancer Center Amsterdam, Amsterdam UMC location AMC, 1105 AZ, Amsterdam, the Netherlands

[7]Department of Epidemiology and Data Science, Amsterdam Public Health, Amsterdam UMC location VUmc, 1081 HV, Amsterdam, the Netherlands

1. Supplementary Figures S1-S24

2. Supplementary Notes 1-2

Markers to identify immune cell types

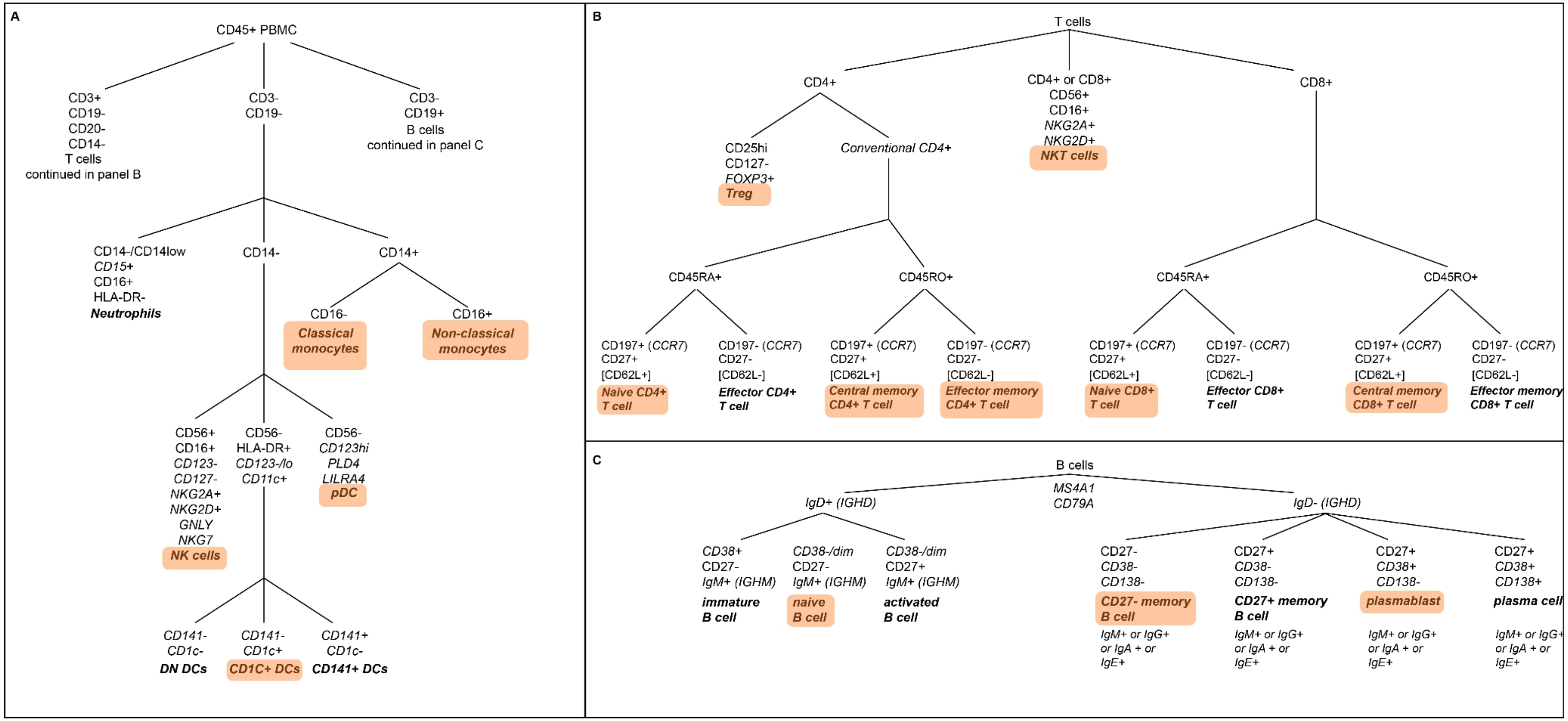

Supplementary Figure S1. A decision tree for phenotyping immune cell populations using CITE-seq data. Panel A corresponds to cells that are negative for CD3 and CD19 markers, which includes Neutrophils, Classical and non-Classical Monocytes, Natural Killer cells (NK), Plasmacytoid dendritic cells (pDC) and three groups of dendritic cells (Double-negative (DN), CD1c+ and CD141+). Panel B corresponds to cells that are positive for CD3+ but negative for CD19, CD20 and CD14. This panel includes regulatory T cells (Treg), Natural Killer T cells (NKT), CD4+ CD45RA+ (Naïve and Effector), CD4+ CD45RO+ (Central Memory and Effector Memory), CD8+ CD45RA+ (Naïve and Effector), CD8+ CD45RO+ (Central Memory and Effector Memory). Panel C corresponds to cells that are positive for CD19 and negative for CD3. This panel includes B cells, positive for IgD (immature, naïve and activated) and negative for IgD (CD27-, CD27+, plasmablast and plasma cells). Highlighted in orange are the cell types found in the two PBMC datasets.

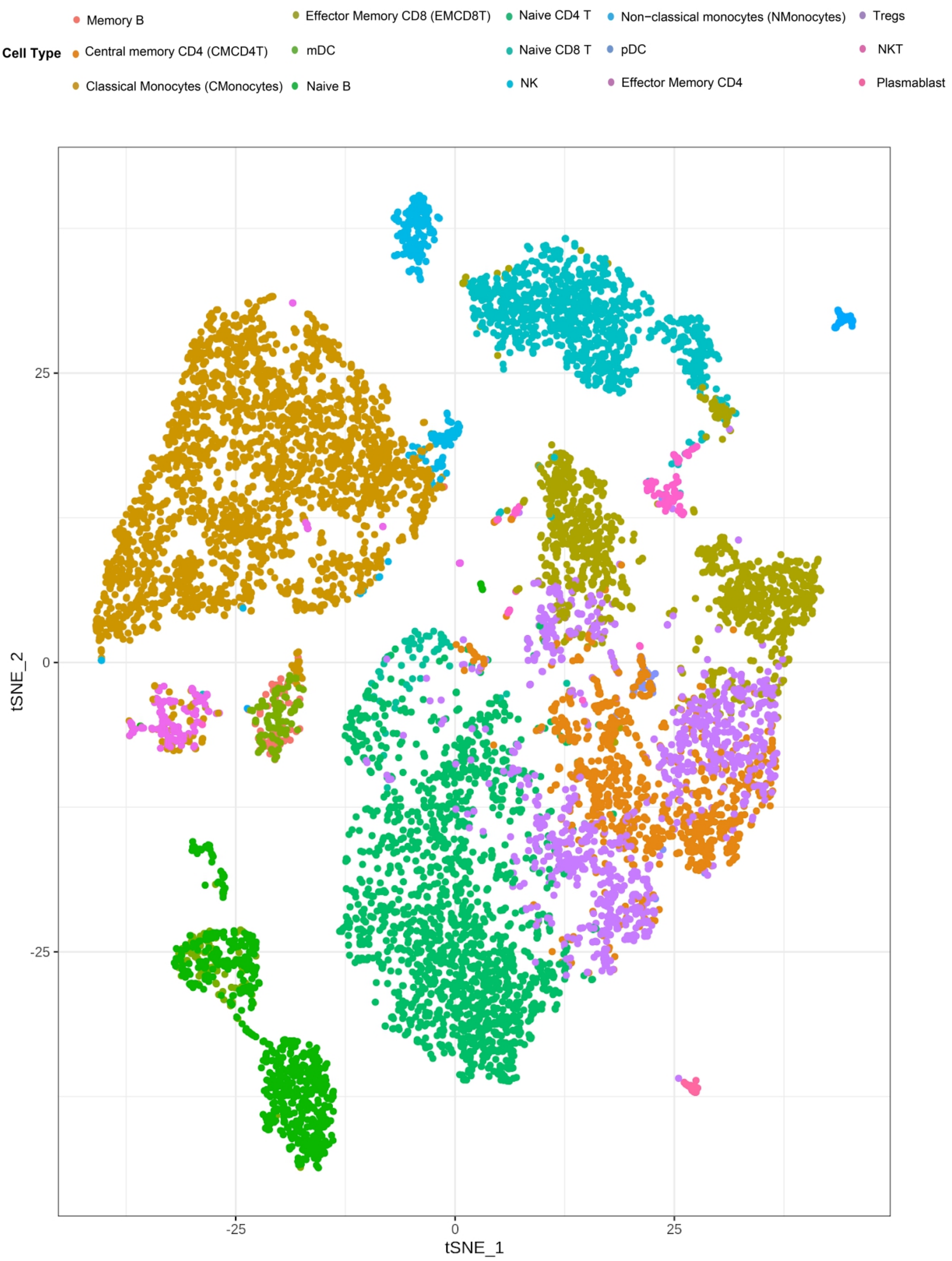

Supplementary Figure S2. Overview of cell populations in the merged PBMC data. t-SNE plot showing the similarities in gene expression profiles between cells in the two PBMC CITE-seq data sets merged. The color of points denotes cell type.

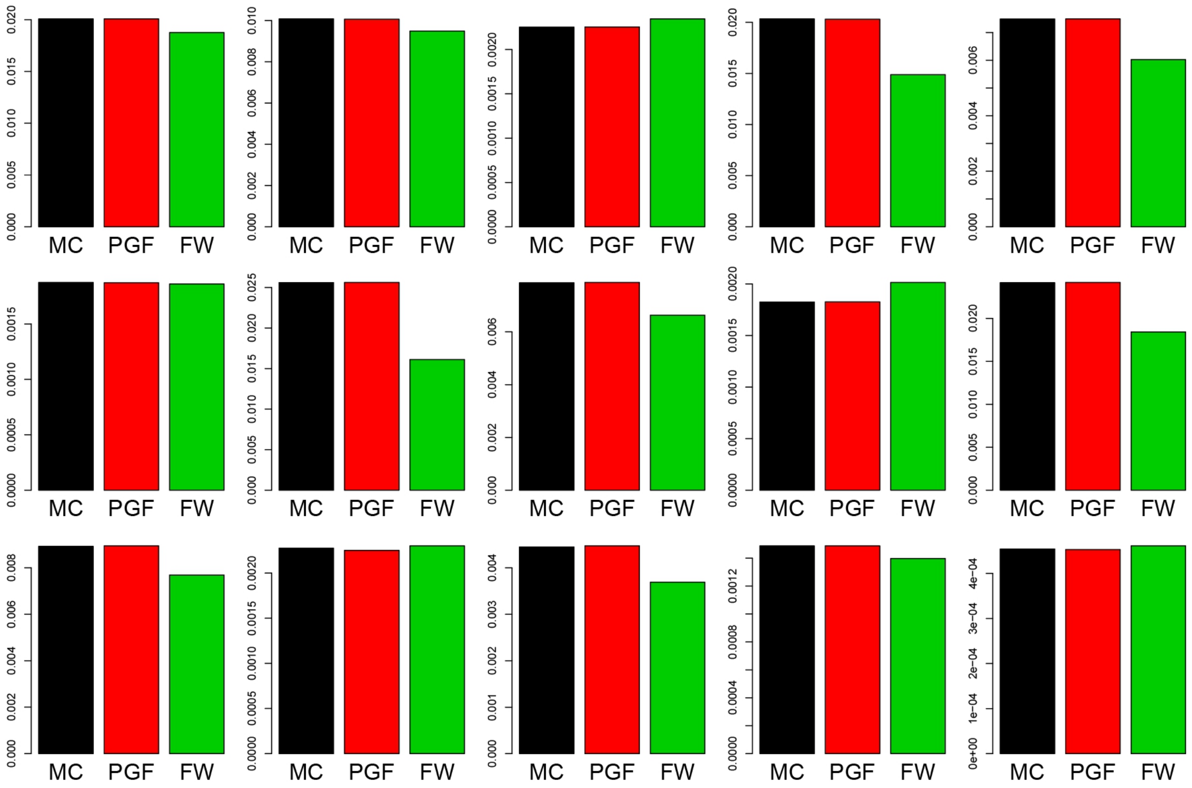

Supplementary Figure S3. Comparison PGF approach with FW-approximation for five simulations and $T = 3$ components. The likelihood is evaluated by probability generation function (PGF; red) approach and Fenton-Wilkinson approximation (FW; green) at $y = \mu_Y/2$ (top row), $y = \mu_Y$ (middle), and $y = 2\mu_Y$ (bottom), with μ_Y is expected value of Y. Monte-Carlo evaluation (MC; black) serves as the baseline.

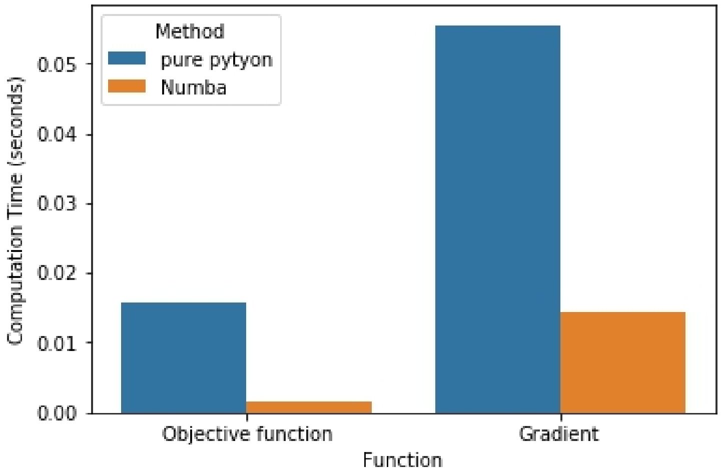

Supplementary Figure S4. Improvement of the computation time by Numba. Computation time (y-axis) of the objective function (left) and gradient (right) used in L-BFGS algorithm of BLADE with (orange) and without (blue) Numba compilation. The data used for evaluation has five cell types, 200 genes, and 50 samples.

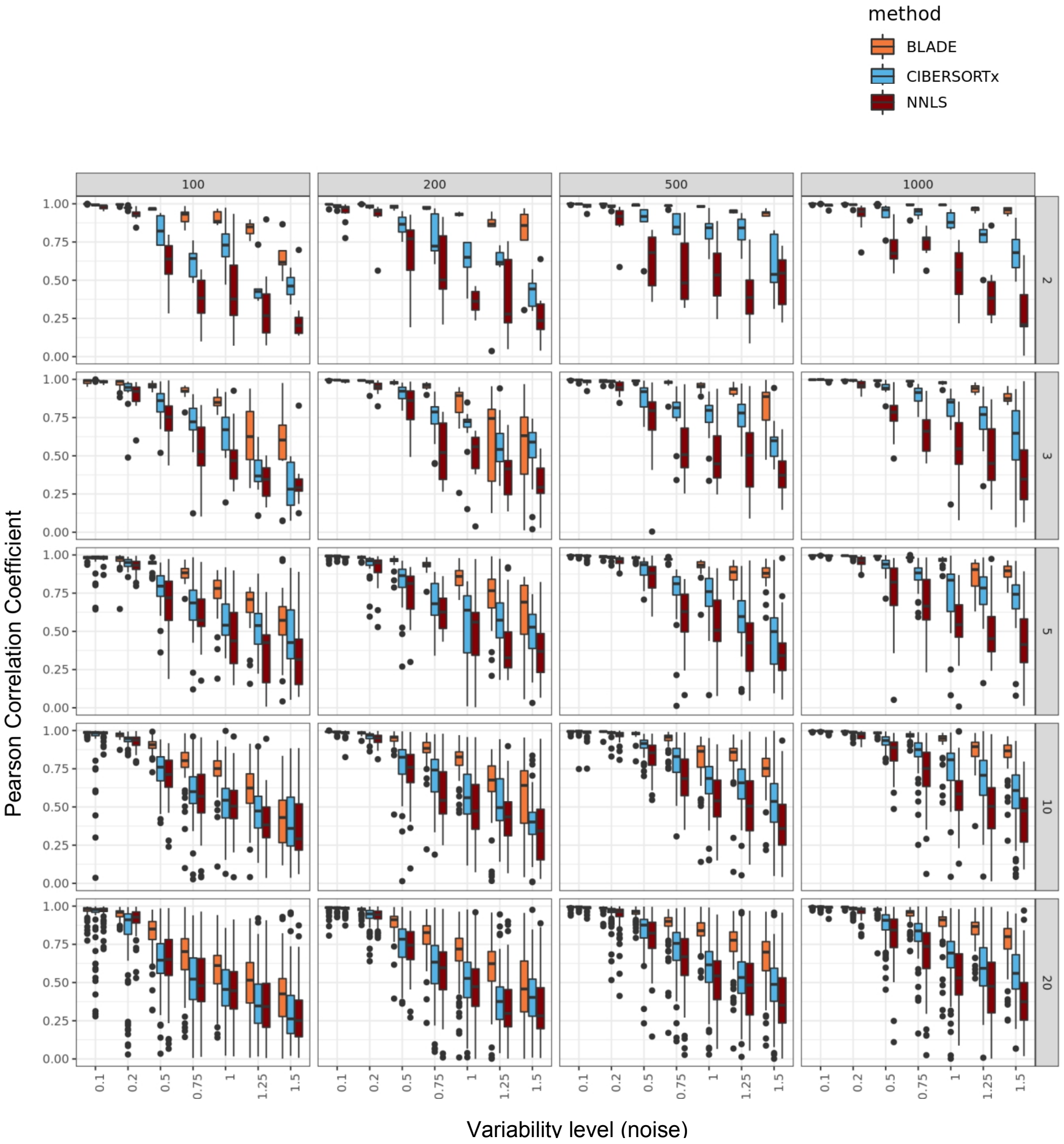

Supplementary Figure S5. Overview of the performance of BLADE and baseline methods in simulation datasets. Performances (Pearson correlation coefficient; y-axis) of BLADE (orange), CIBERSORTx (blue), and NNLS (dark red) in predicting cellular fraction of simulation data with diverse variability levels (standard deviation of 0.1-1.5; x-axis), number of cells (2-20 cell types; rows), and number of genes (100-1000 genes; columns). Each boxplot shows the performance from 5 independent data sets for the corresponding number of cell types (i.e., n=5 times the number of cell types defined in the row). The standard boxplot notation was used (lower/upper hinges - first/third quartiles; whiskers extend from the top/bottom hinges to the largest/lowest values no further than 1.5 * inter-quartile ranges).

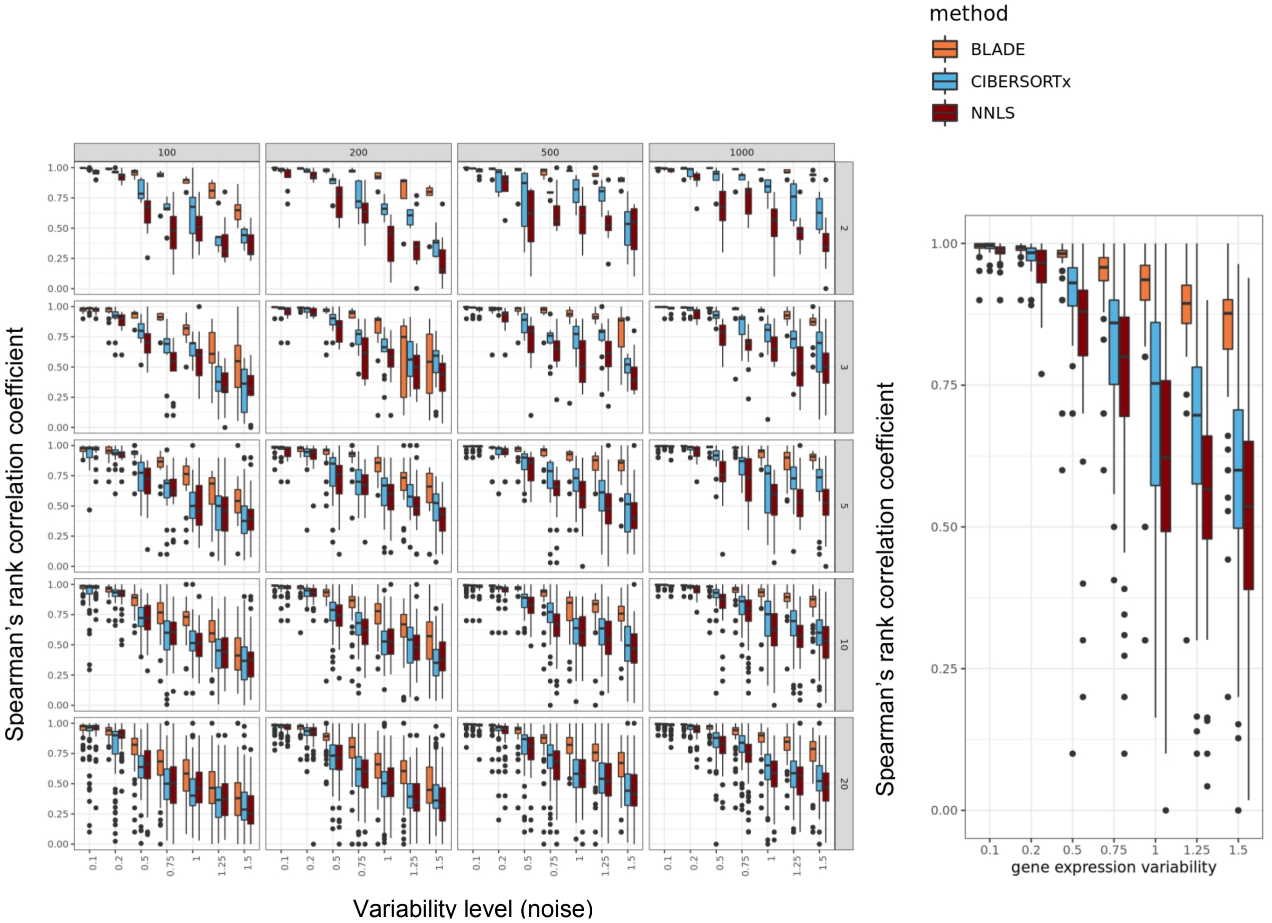

Supplementary Figure S6. Overview of the performance in Spearman's rank correlation of BLADE and baseline methods in simulation datasets. Performances (Spearman's rank correlation coefficient; y-axis) of BLADE (orange), CIBERSORTx (blue), and NNLS (dark red) in predicting cellular fraction of simulation data with diverse variability levels (standard deviation of 0.1-1.5; x-axis), number of cells (2-20 cell types; rows; 10 cell type dataset highlighted on the right), and number of genes (100-1000 genes; columns; 1000 genes dataset highlighted on the right). Each boxplot shows the performance from 5 independent data sets for the corresponding number of cell types (i.e., n=5 times the number of cell types defined in the row). The standard boxplot notation was used (lower/upper hinges - first/third quartiles; whiskers extend from the top/bottom hinges to the largest/lowest values no further than 1.5 * inter-quartile ranges).

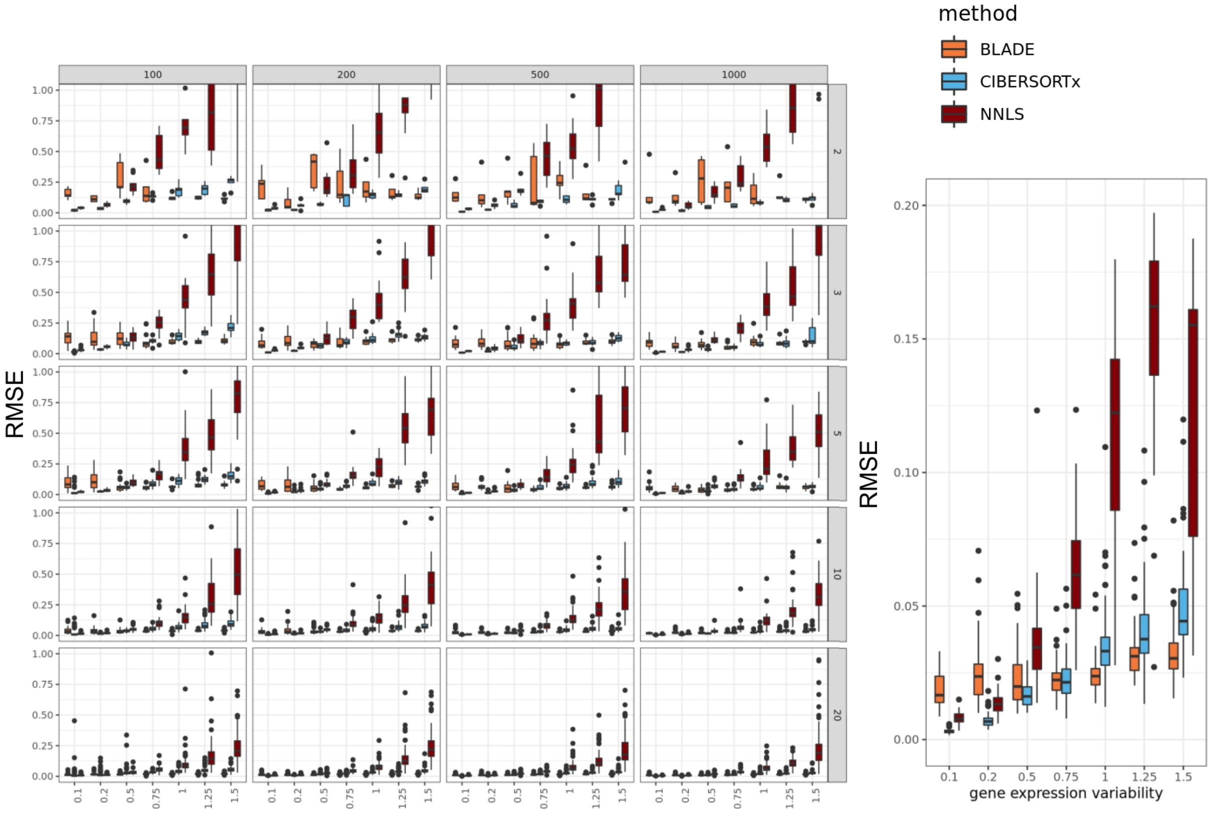

Supplementary Figure S7. Overview of the performance in root mean square error (RMSE) of BLADE and baseline methods in simulation datasets. Performances (root mean square error; RMSE; y-axis) of BLADE (orange), CIBERSORTx (blue), and NNLS (dark red) in predicting cellular fraction of simulation data with diverse variability levels (standard deviation of 0.1-1.5; x-axis), number of cells (2-20 cell types; rows; 10 cell type dataset highlighted on the right), and number of genes (100-1000 genes; columns; 1000 genes dataset highlighted on the right). Each boxplot shows the performance from 5 independent data sets for the corresponding number of cell types (i.e., n=5 times the number of cell types defined in the row). The standard boxplot notation was used (lower/upper hinges - first/third quartiles; whiskers extend from the top/bottom hinges to the largest/lowest values no further than 1.5 * inter-quartile ranges). Note that RMSE is not meant to be compared between data set with the different number of cell types, as it depends a lot on the abundance of cell types. (according to RMSE, the performance gets better with the higher number of cell types, which is misleading).

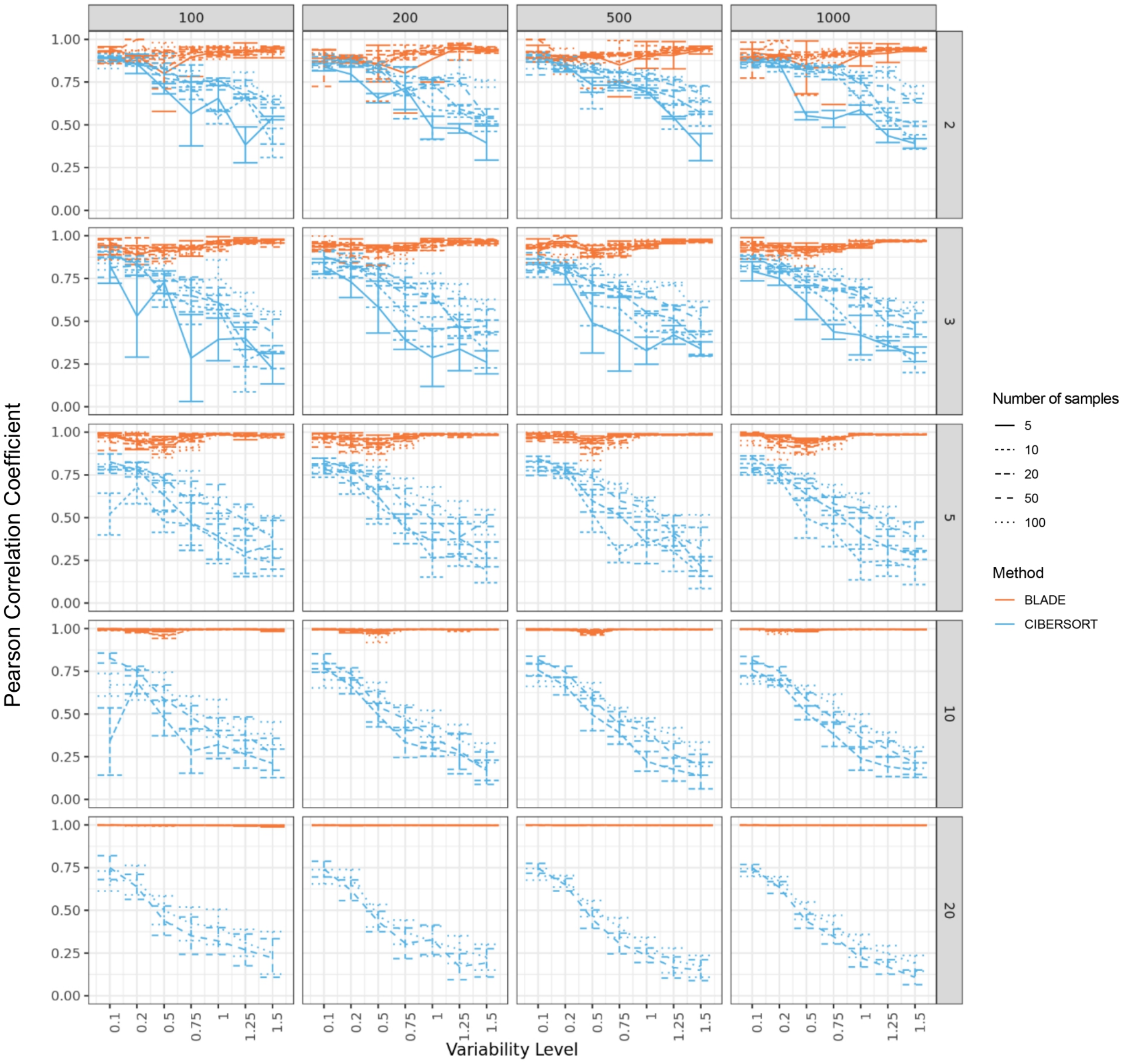

Supplementary Figure S8. Overview of the performance in group mode purification of BLADE and CIBERSORTx in simulation datasets. Performances (Pearson correlation coefficient; y-axis) of BLADE (orange) and CIBERSORTx (blue) in predicting gene expression profiles per cell type (group mode) of simulation data with diverse variability levels (standard deviation of 0.1-1.5; x-axis), number of cells (2-20 cell types; rows), number of genes (100-1000 genes; columns), and number of samples (5-100 samples; denoted by the type of line). Each point in line plots shows the average and standard deviation of the Pearson correlation coefficient of all cell types (n=2-20) in the corresponding data set.

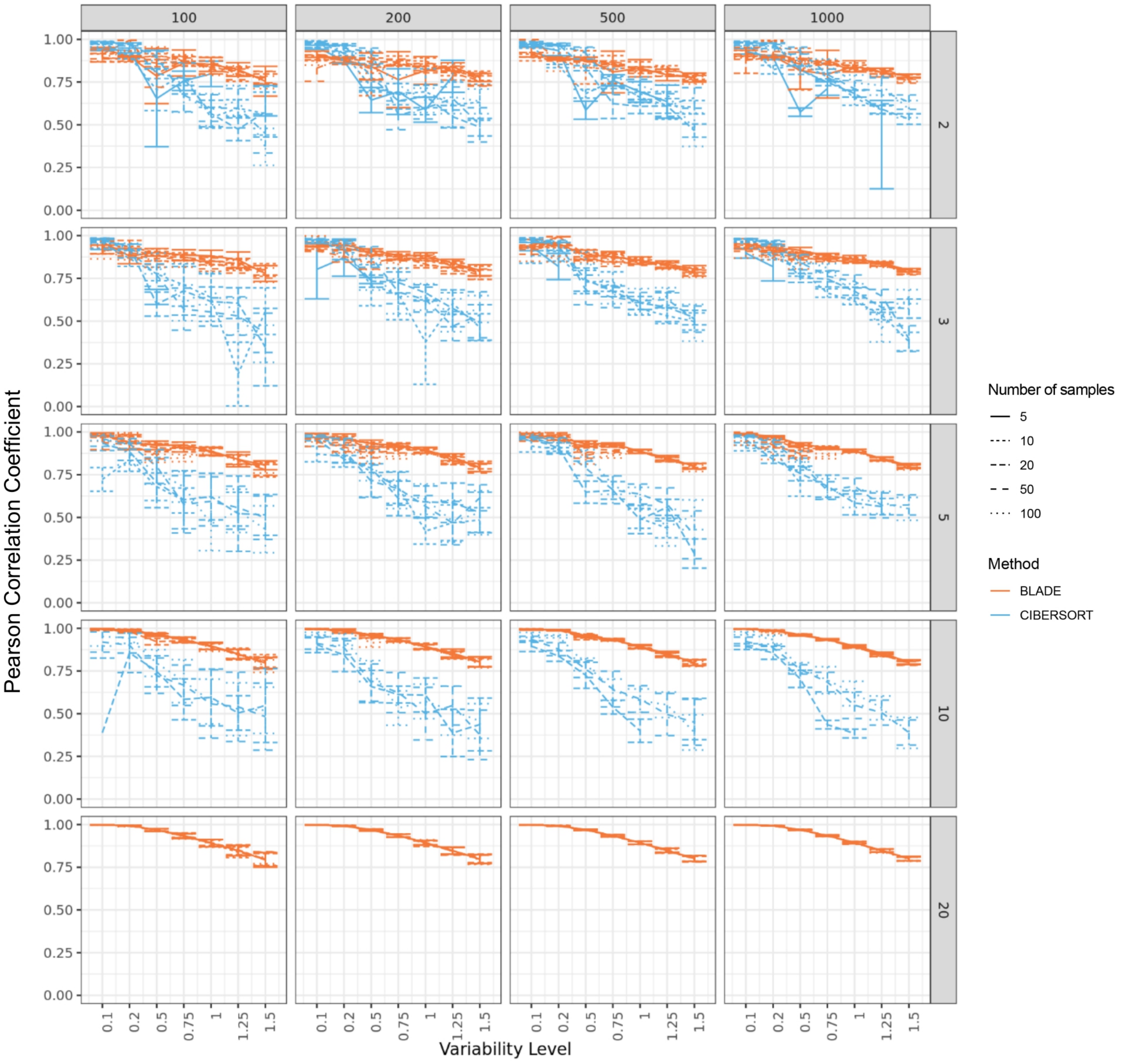

Supplementary Figure S9. Overview of the performance in high-resolution mode purification of BLADE and CIBERSORTx in simulation datasets. Performances (Pearson correlation coefficient; y-axis) of BLADE (orange) and CIBERSORTx (blue) in predicting gene expression profiles per cell type per sample (high-resolution mode) of simulation data with diverse variability levels (standard deviation of 0.1-1.5; x-axis), number of cells (2-20 cell types; rows), number of genes (100-1000 genes; columns), and number of samples (5-100 samples; denoted by the type of line). Each point in line plots shows the average and standard deviation of the Pearson correlation coefficient of all pairs (n=10-200) of cell types (n=2-20) and samples (n=5-100) in the corresponding data set.

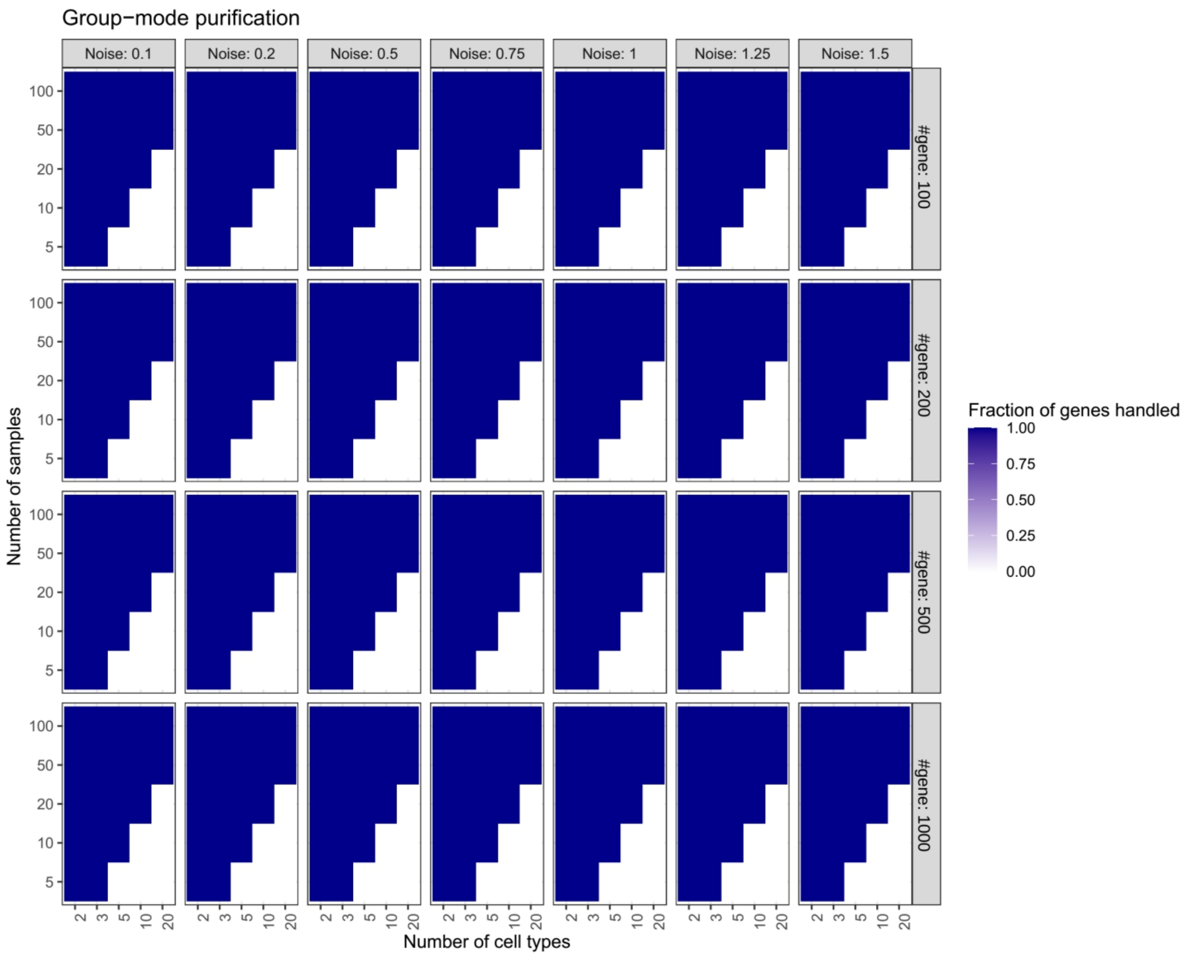

Supplementary Figure S10. Overview of missing values in group mode purification of CIBERSORTx in simulation datasets. Missing values of CIBERSORTx in group mode purification for simulation data with diverse settings (number of genes: rows; variability levels: columns; number of cell types: x-axis; number of samples: y-axis).

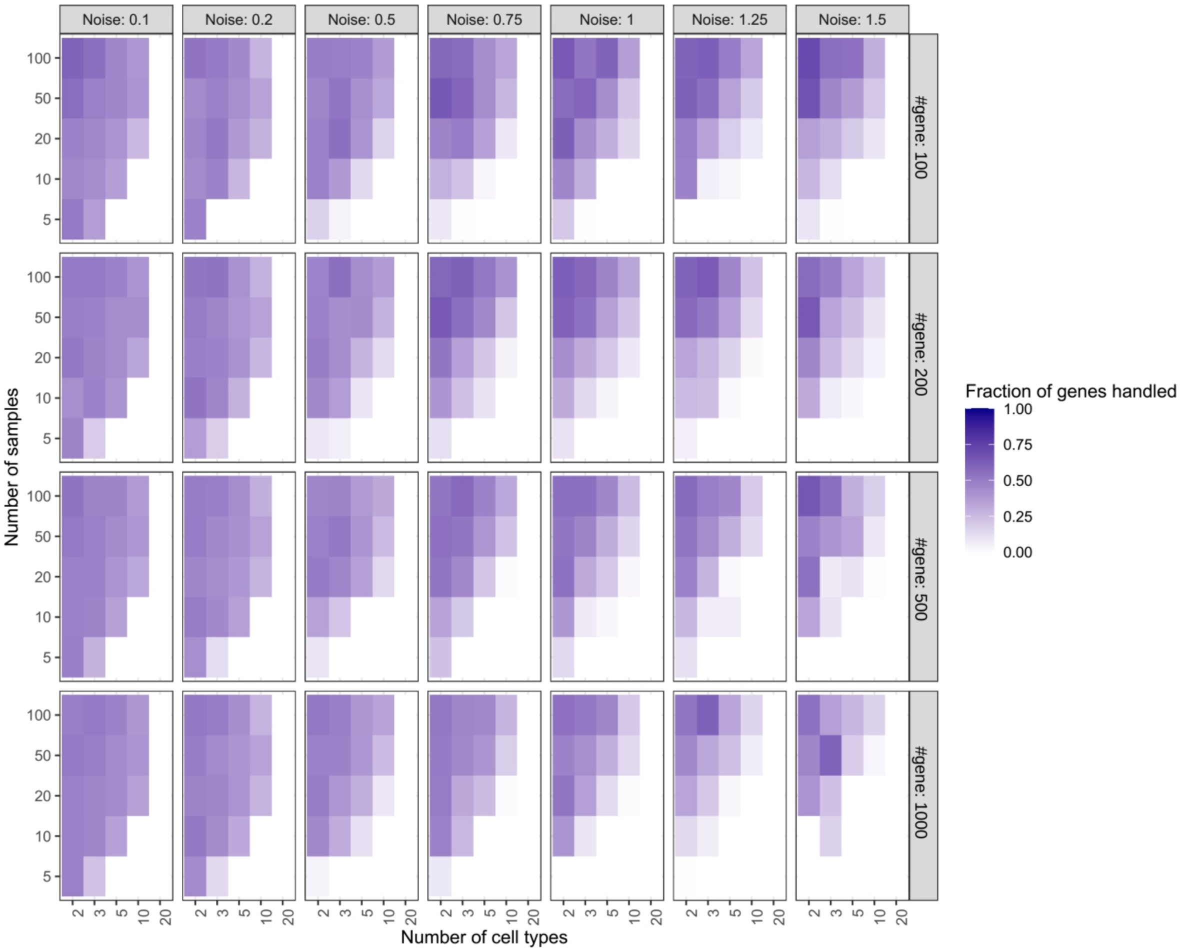

Supplementary Figure S11. Overview of missing values in high-resolution mode purification of CIBERSORTx in simulation datasets. Missing values of CIBERSORTx in high-resolution mode purification for simulation data with diverse settings (number of genes: rows; variability levels: columns; number of cell types: x-axis; number of samples: y-axis).

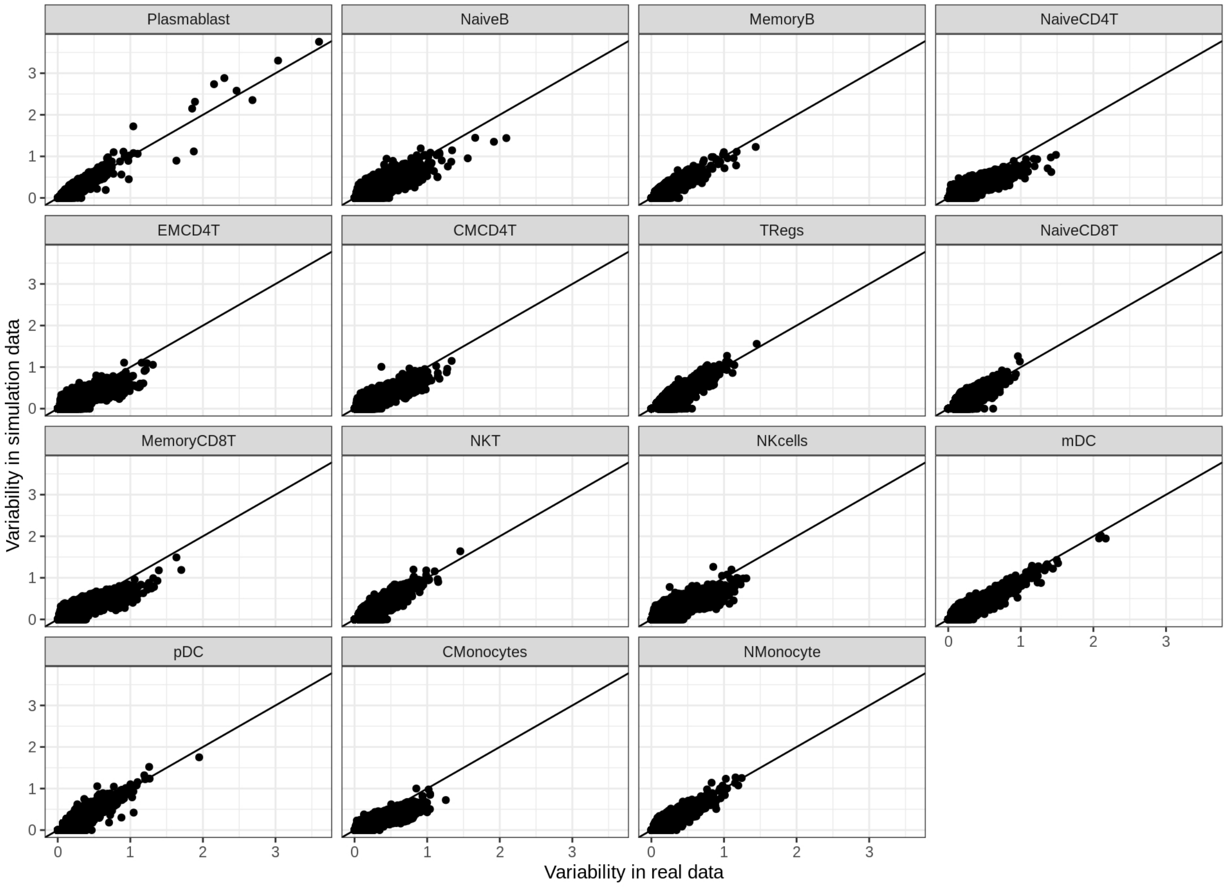

Supplementary Figure S12. Overview of variability in cell-type-specific gene expression profiles of simulated and real PBMC data. Scatter plots show the variability of 1,007 genes in PBMC simulation data (y-axis) in the 15 cell types (grids) recapitulates the one observed in real data (x-axis).

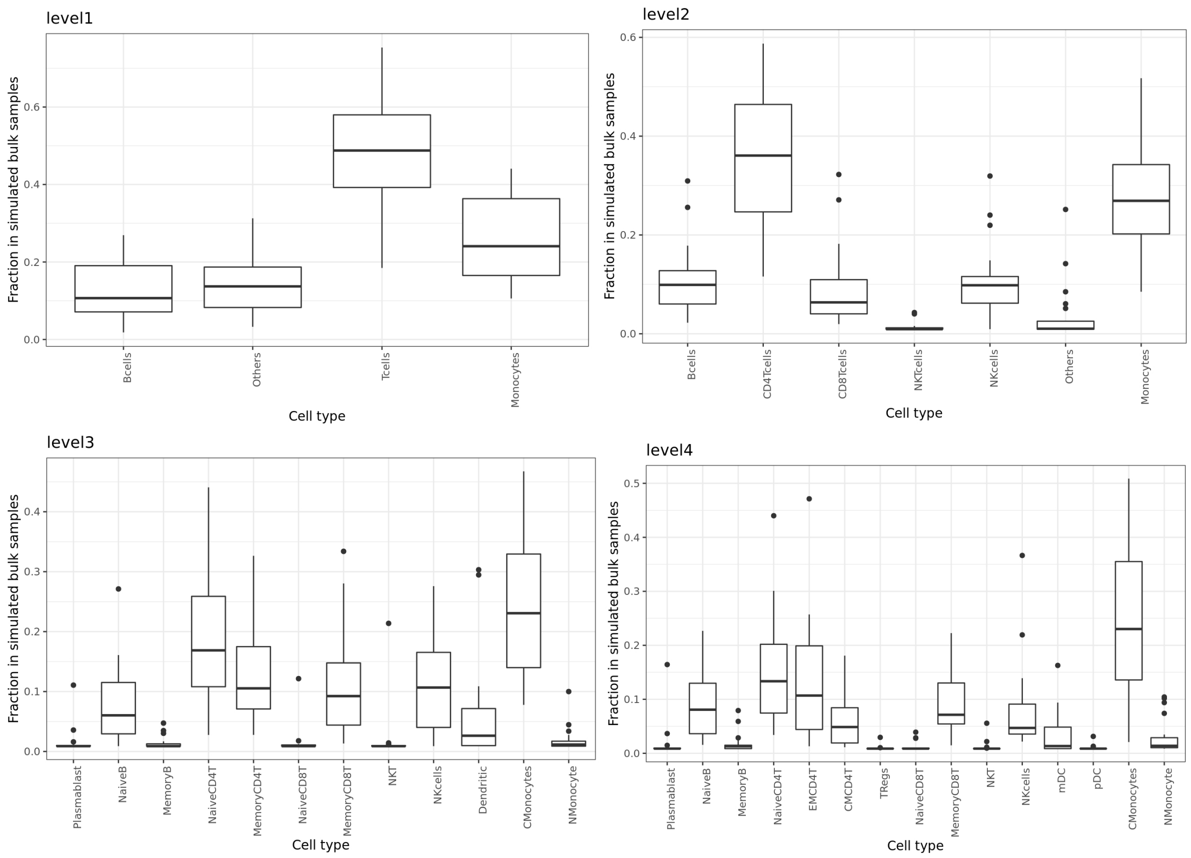

Supplementary Figure S13. Fraction of cell types in the four different levels of PBMC simulation data. The fraction (y-axis) of cell types (x-axis) in 20 simulated PBMC datasets in levels 1-4 (each panel). The standard boxplot notation was used (lower/upper hinges - first/third quartiles; whiskers extend from the top/bottom hinges to the largest/lowest values no further than 1.5 * inter-quartile ranges).

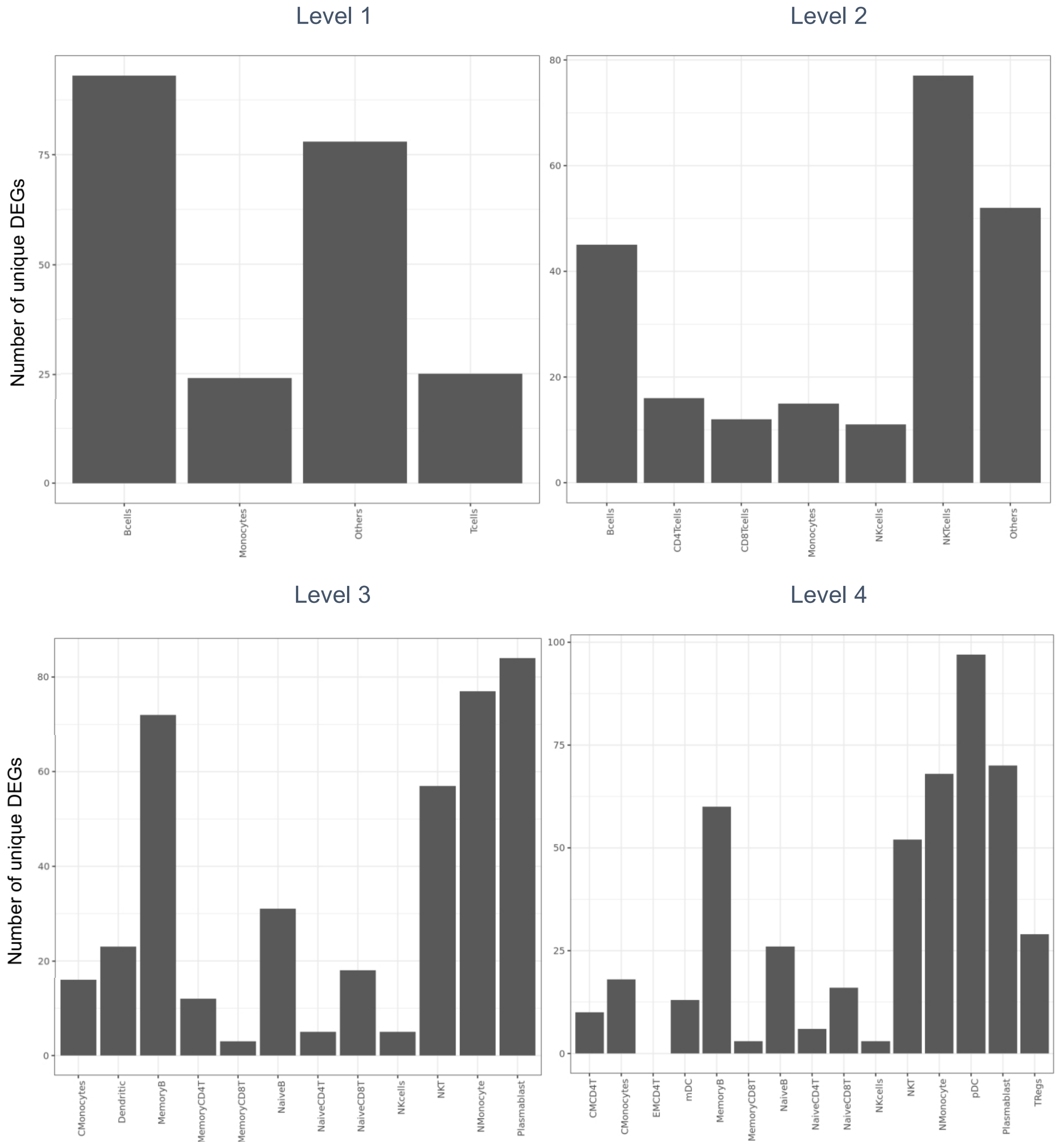

Supplementary Figure S14. The number of unique differentially expressed genes per cell type in the four different levels of PBMC data. The number of unique differentially expressed genes (DEGs; y-axis) in the PBMC cell types (x-axis), classified by four different levels.

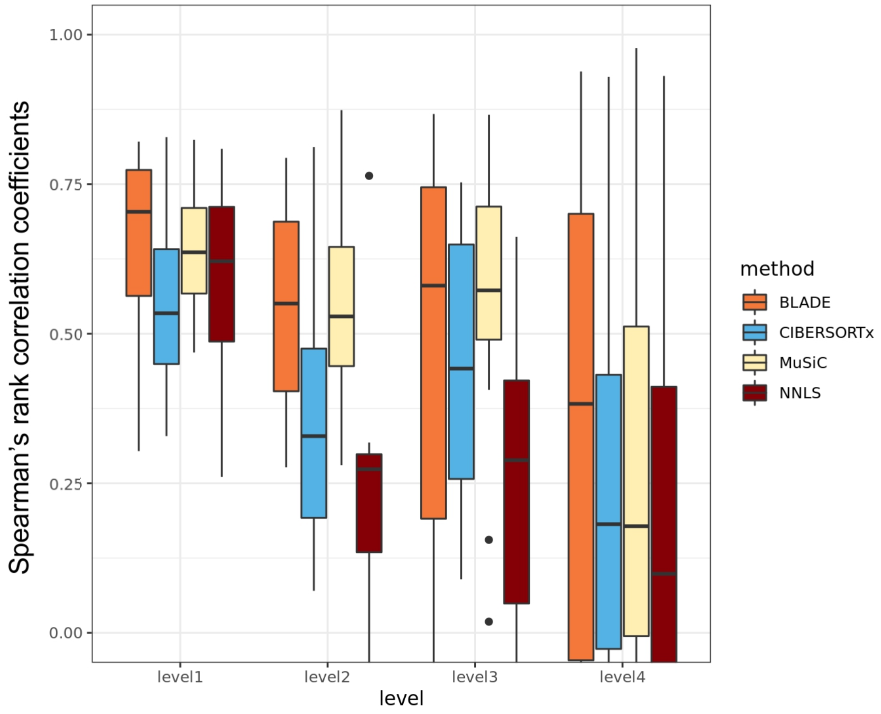

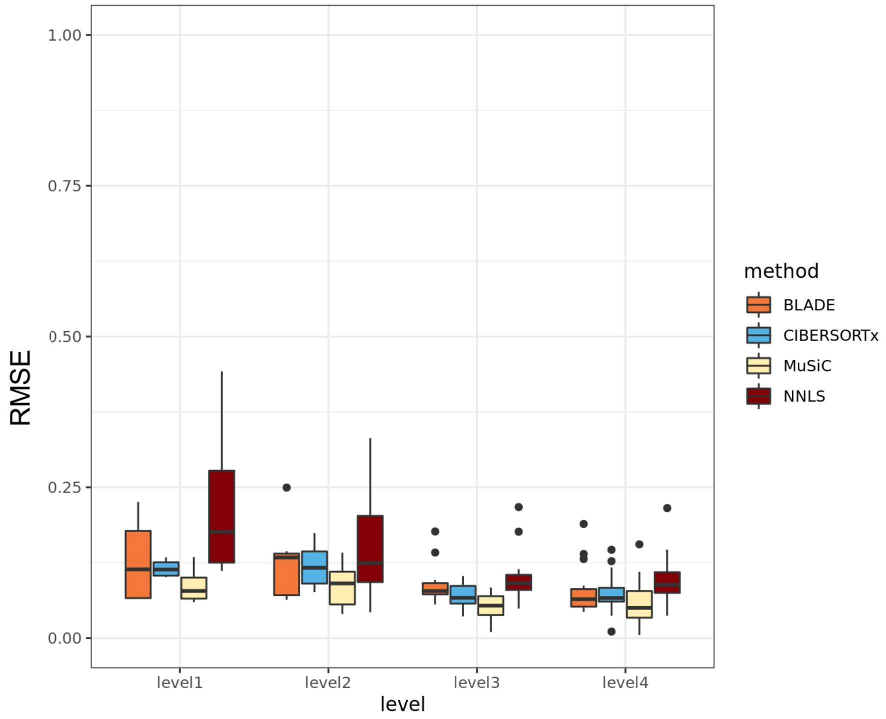

Supplementary Figure S15. Performance of BLADE in alternative metrics compared to baseline methods in fraction estimation of simulated PBMC data. Spearman's rank correlation coefficient (y-axis; top) and RMSE (y-axis; bottom) compared between BLADE (orange) and baseline methods (CIBERSORTx (blue), MuSiC (light yellow), and NNLS (dark red)) for level 1-4 PBMC data (x-axis). Note that RMSE is not meant to be compared between data set with the different number of cell types, as it depends a lot on the abundance of cell types. (according to RMSE, the performance gets better with the higher number of cell types, which is misleading). The standard boxplot notation was used (lower/upper hinges - first/third quartiles; whiskers extend from the top/bottom hinges to the largest/lowest values no further than 1.5 * inter-quartile ranges).

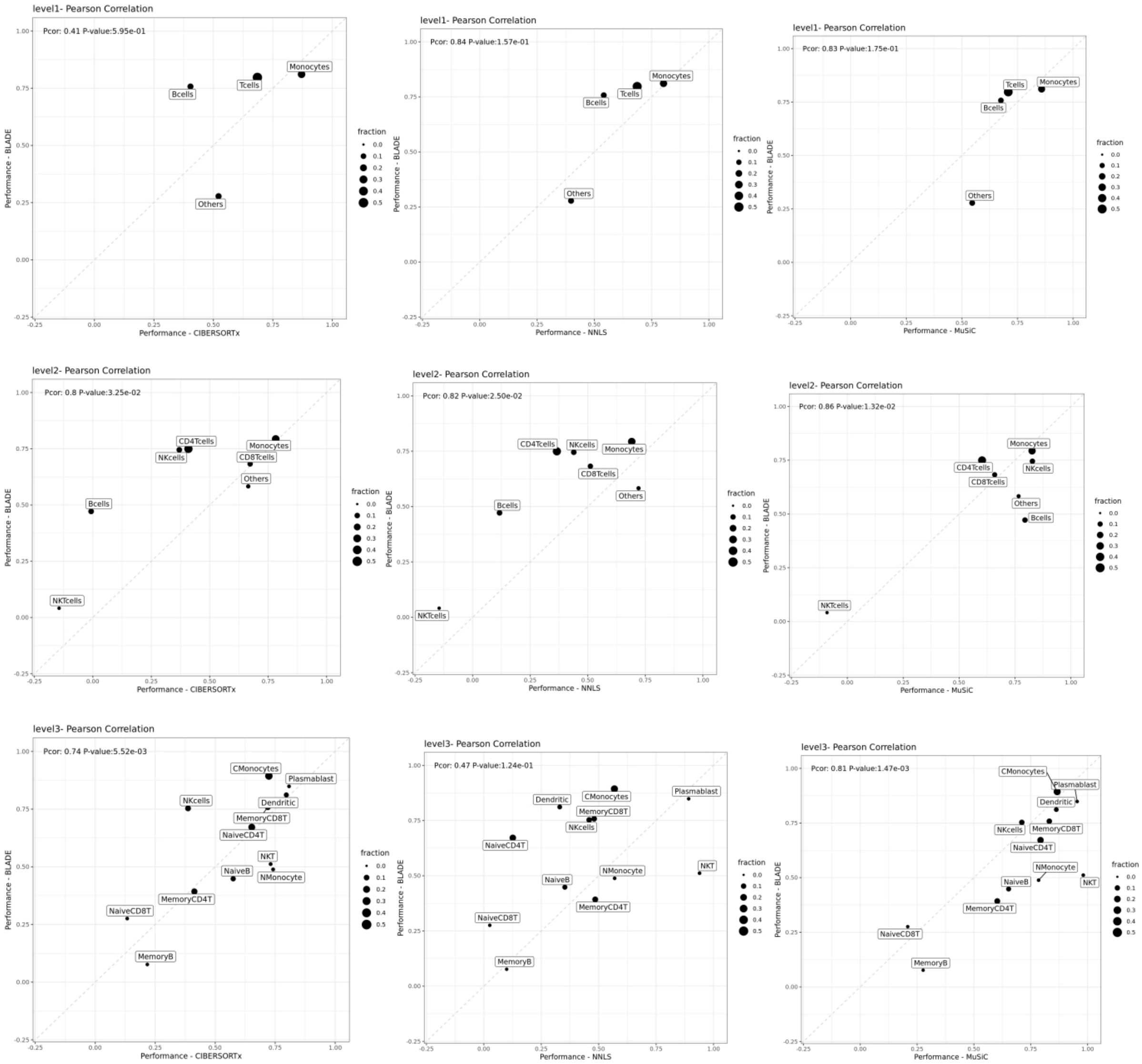

Supplementary Figure S16. Performance of BLADE compared to baseline methods in levels 1-3 PBMC simulation data. Pearson Correlation coefficient compared between BLADE (y-axis) and baseline methods (CIBERSORTx(left), NNLS (middle), and MuSiC (right)) for level 1-3 PBMC dataset (top-bottom rows). Pearson correlation coefficient and two-tailed test P-values are indicated at the top left in each panel.

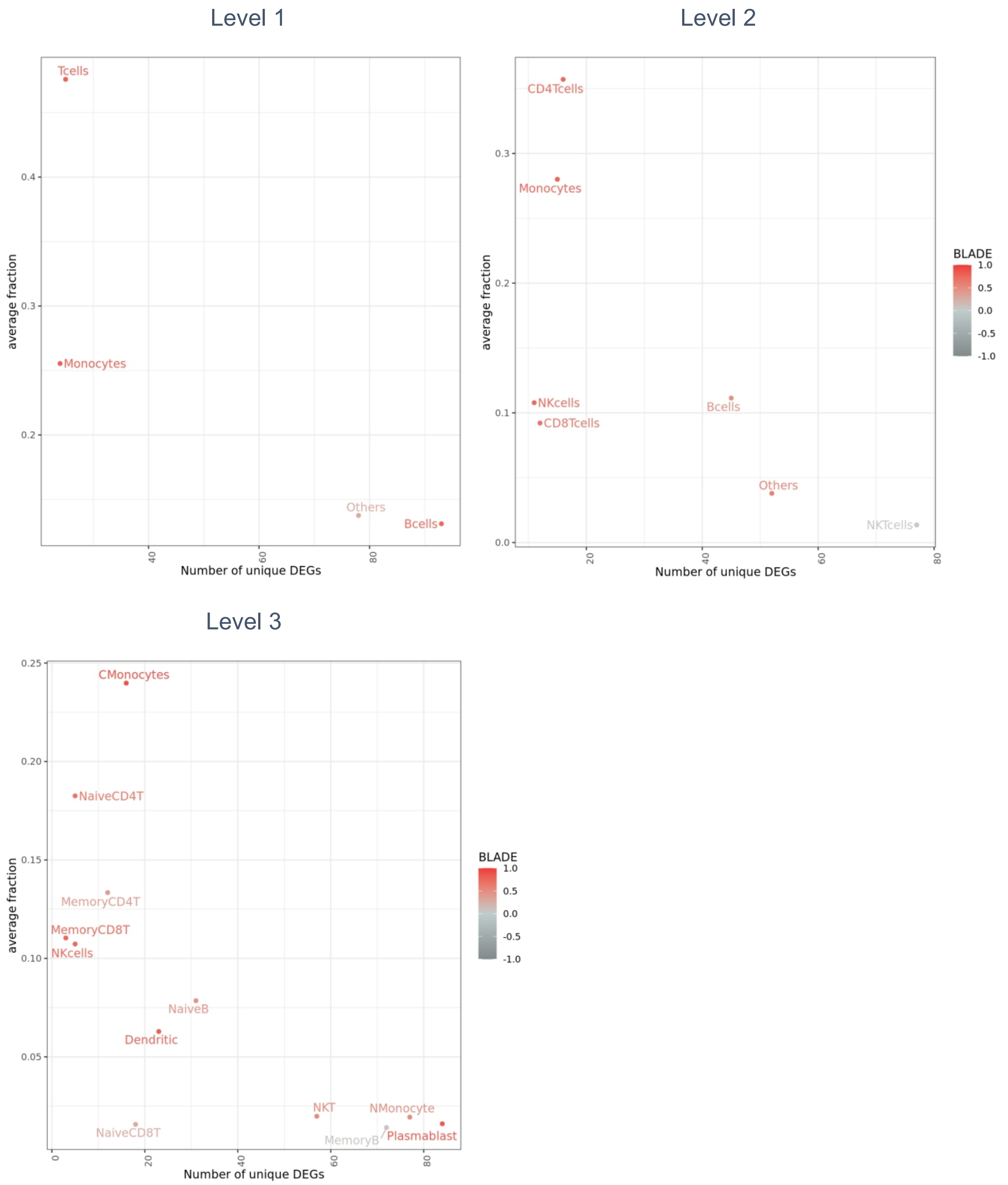

Supplementary Figure S17. Association between BLADE performance, the abundance of cell types, and the number of unique DEGs. Average fraction (y-axis) and the number of unique deferentially expressed genes (x-axis) of each cell type in levels 1-3 of PBMC data (levels 1-3 at the top, right, and bottom). The color of dots denotes the performance of BLADE measured by the Pearson correlation coefficient.

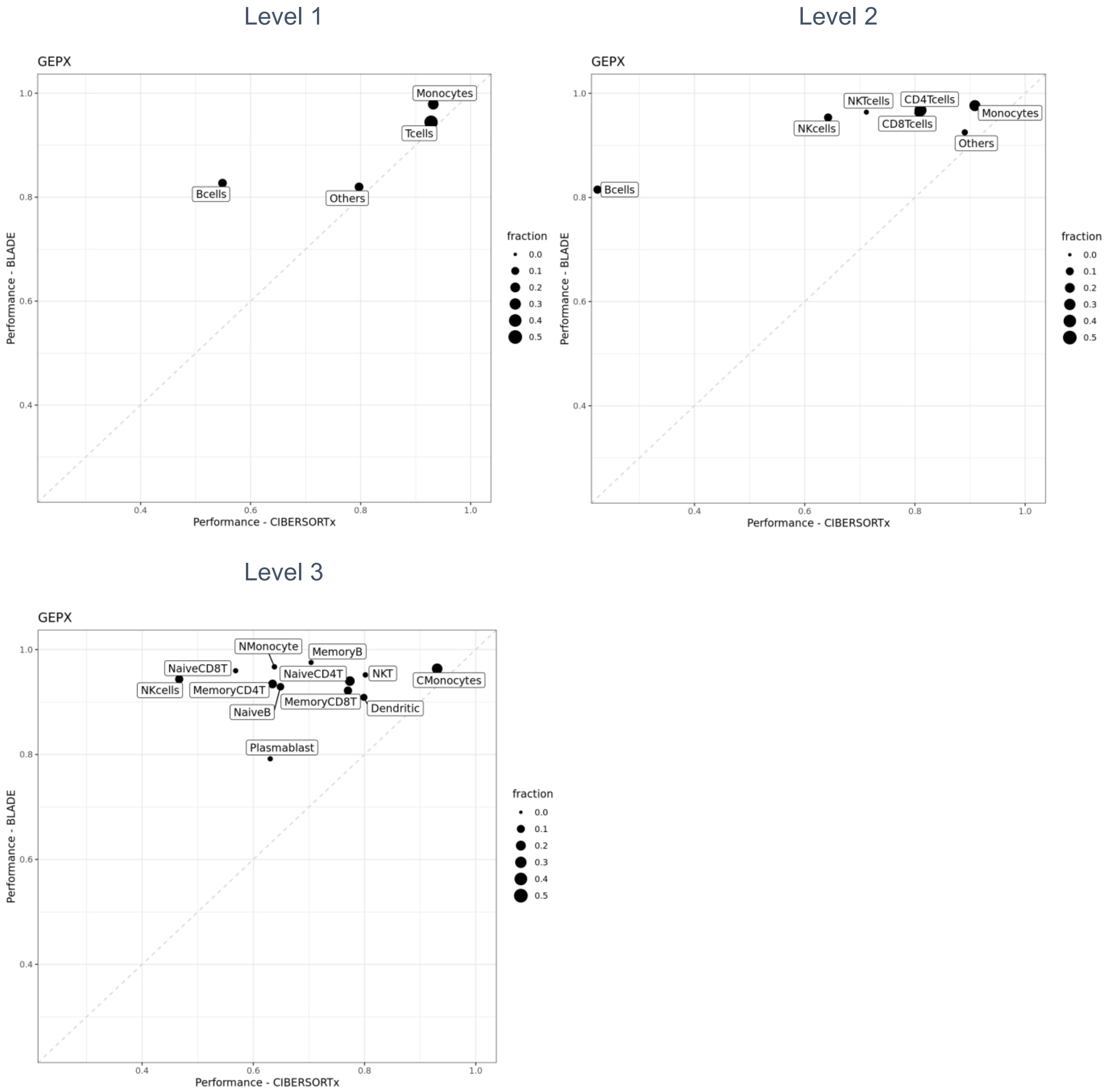

Supplementary Figure S18. Comparison between BLADE and CIBERSORTx for group mode purification in PBMC simulation dataset. Pearson correlation coefficient of BLADE (y-axis) and CIBERSORTx (x-axis) in estimating cell type-specific gene expression profiles (group-mode purification) in level 1-3 PBMC simulation data (left, right, and bottom panel).

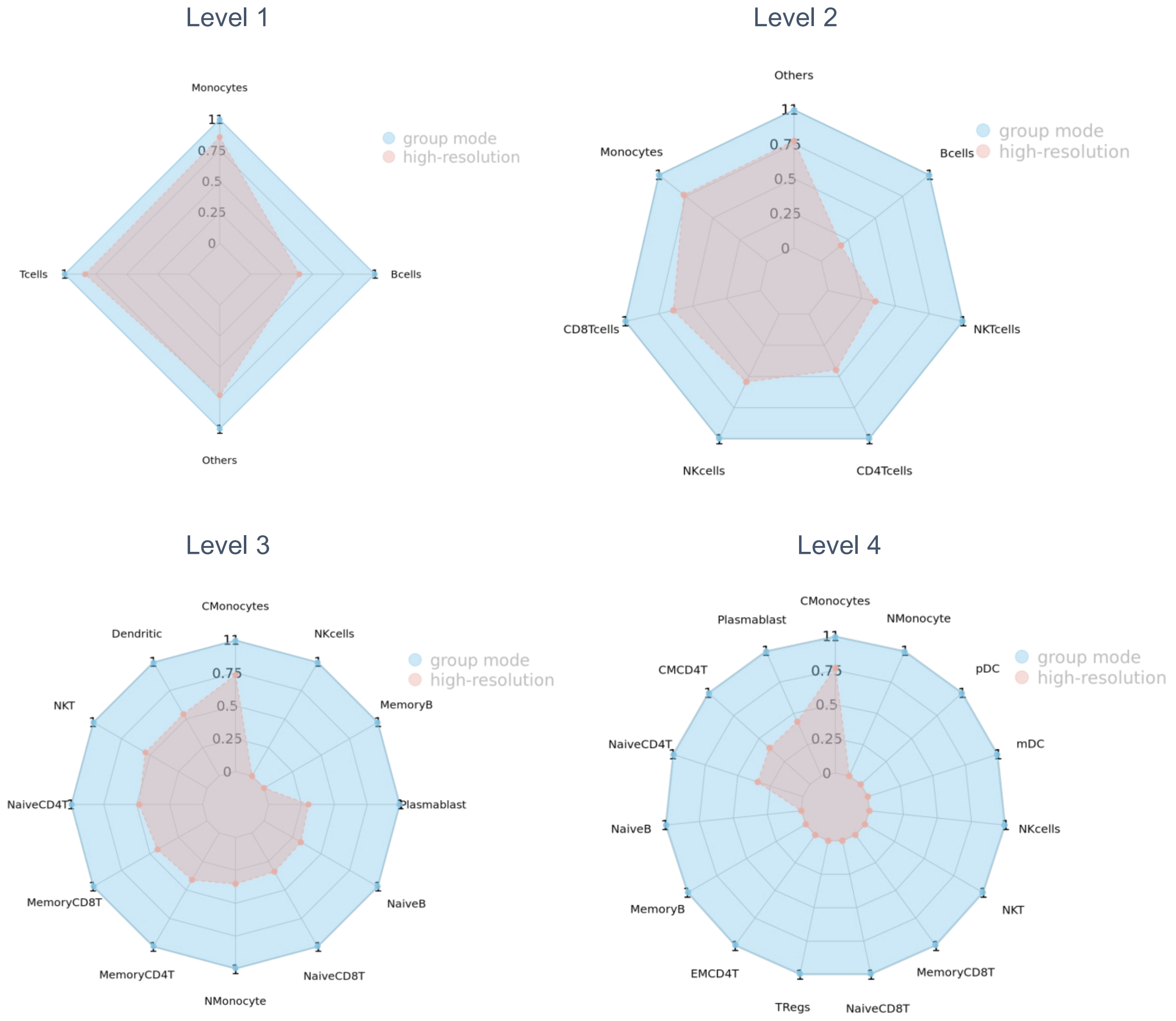

Supplementary Figure S19. Fraction of genes purified by CIBERSORTx in PBMC simulation data. Radar plots represent the gene fraction with estimated cell-type-specific gene expression profiles for each cell type in group mode (blue) and high-resolution mode (red) in level 1-4 PBMC simulation data.

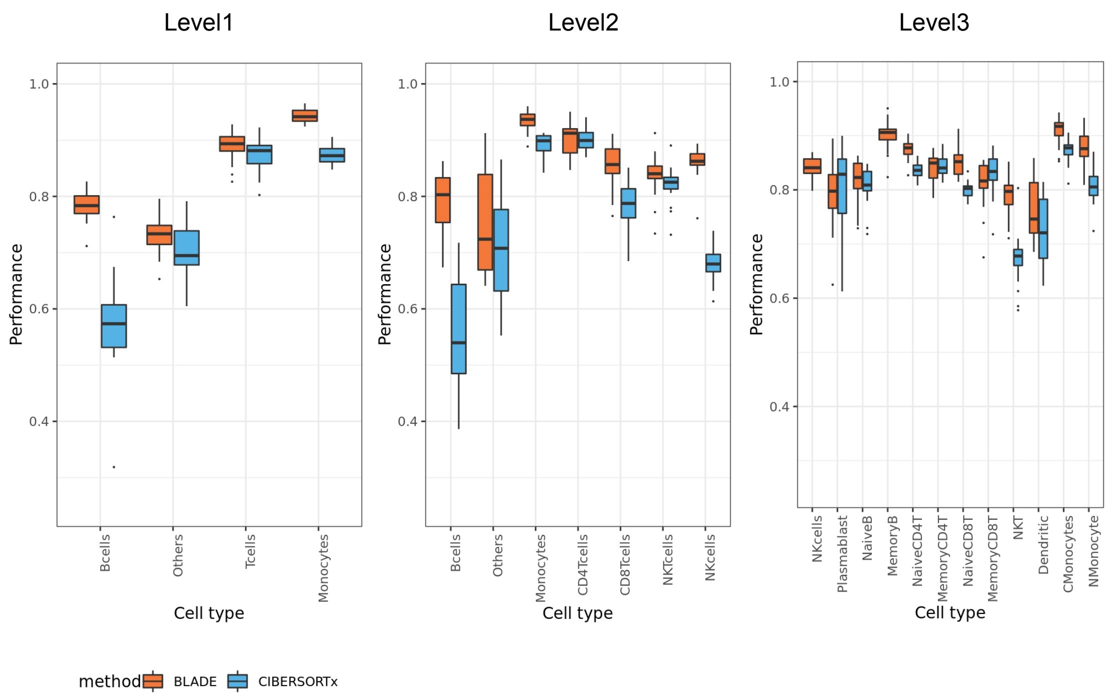

Supplementary Figure S20. Performance of BLADE in high-resolution mode purification for simulated PBMC bulk RNA-seq data. Performance (Pearson correlation coefficient; y-axis) of BLADE (orange) and CIBERSORTx (blue) in estimating gene expression profiles per cell type (x-axis) and per sample in levels 1-3 (n=20 samples per cell type).

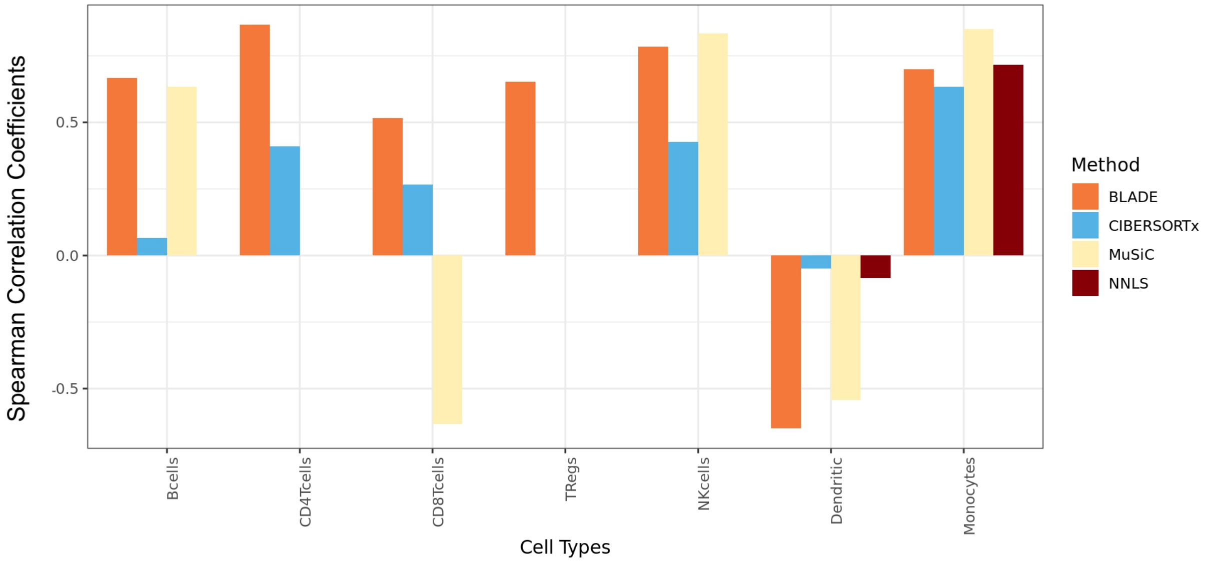

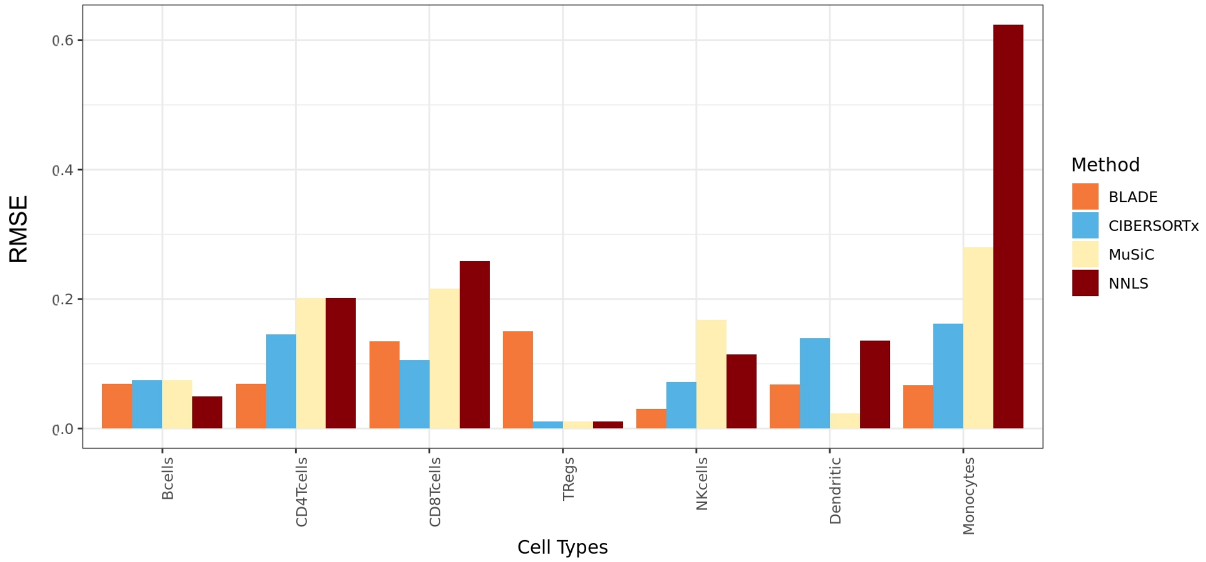

Supplementary Figure S21. Alternative performance metrics for deconvolution of bulk RNA-seq data of PBMC immune cell mixtures. Performances in Spearman correlation (y-axis; top) and root mean squared error (RMSE; y-axis; bottom) of BLADE (orange), CIBERSORTx (blue), MuSiC (light yellow), and NNLS (dark red) in estimating the cell fractions in all samples of each cell type (x-axis) determined by flow cytometry.

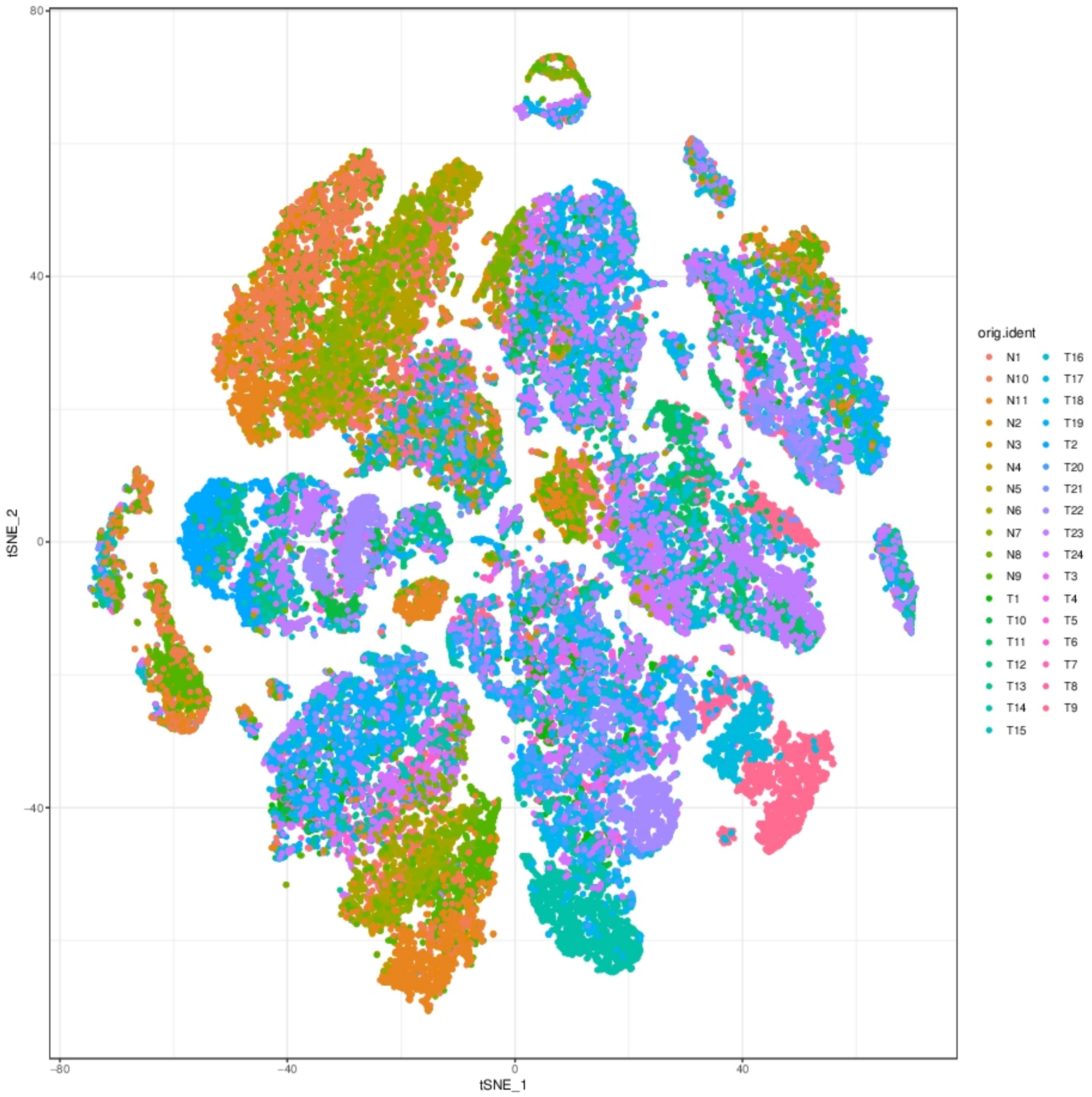

Supplementary Figure S22. Overview of cells in PDAC single-cell RNAseq data. T-sne plot represents the similarity in gene expression profiles among cells in the PDAC scRNA-seq data. The color indicates normal pancreas (N1-N11) and PDAC samples (T1-T24).

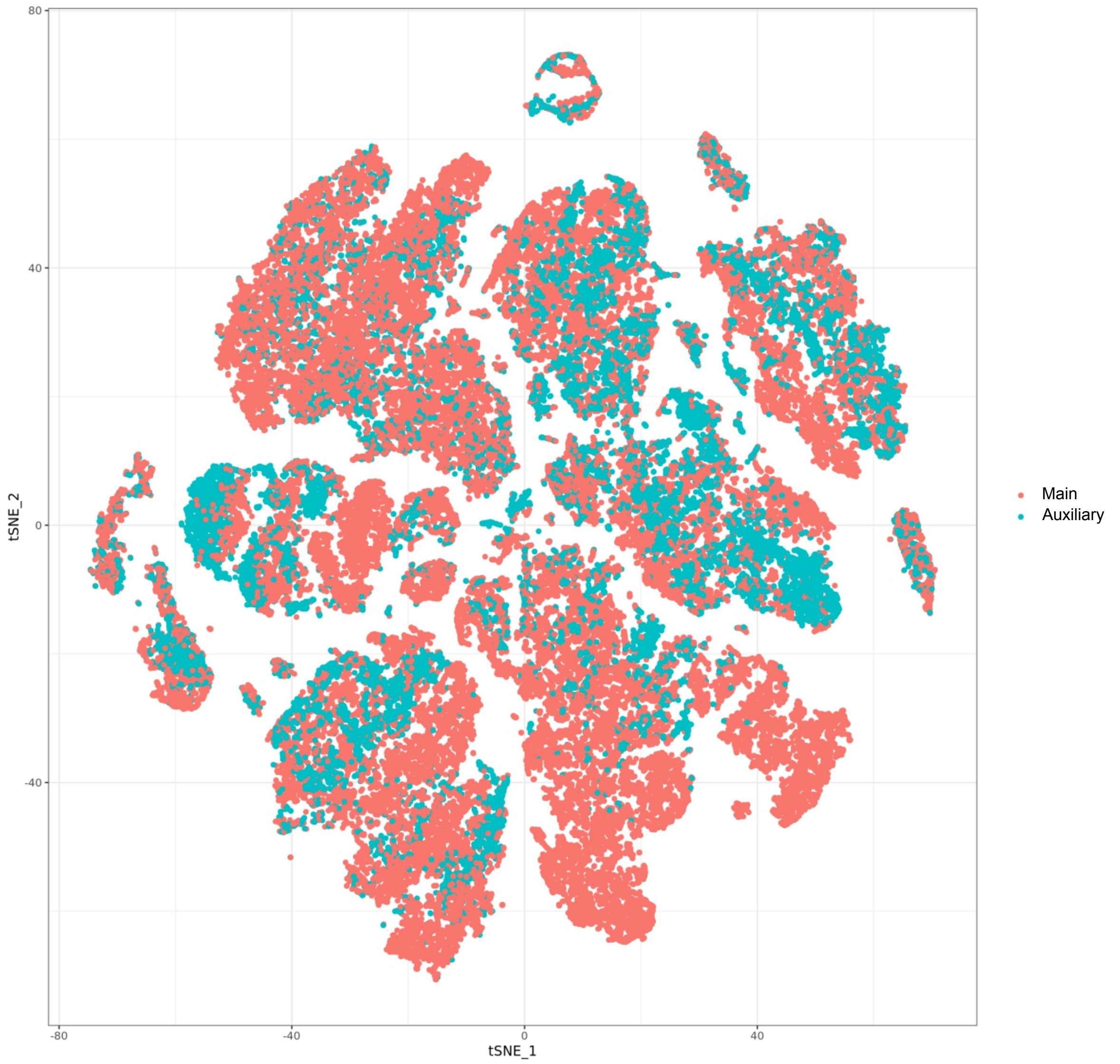

Supplementary Figure S23. Overview of cells in PDAC single-cell RNAseq data. T-sne plot represents the similarity in gene expression profiles among cells in the PDAC scRNA-seq data. The color indicates auxiliary (n=6; blue) and main (n=29; red) samples.

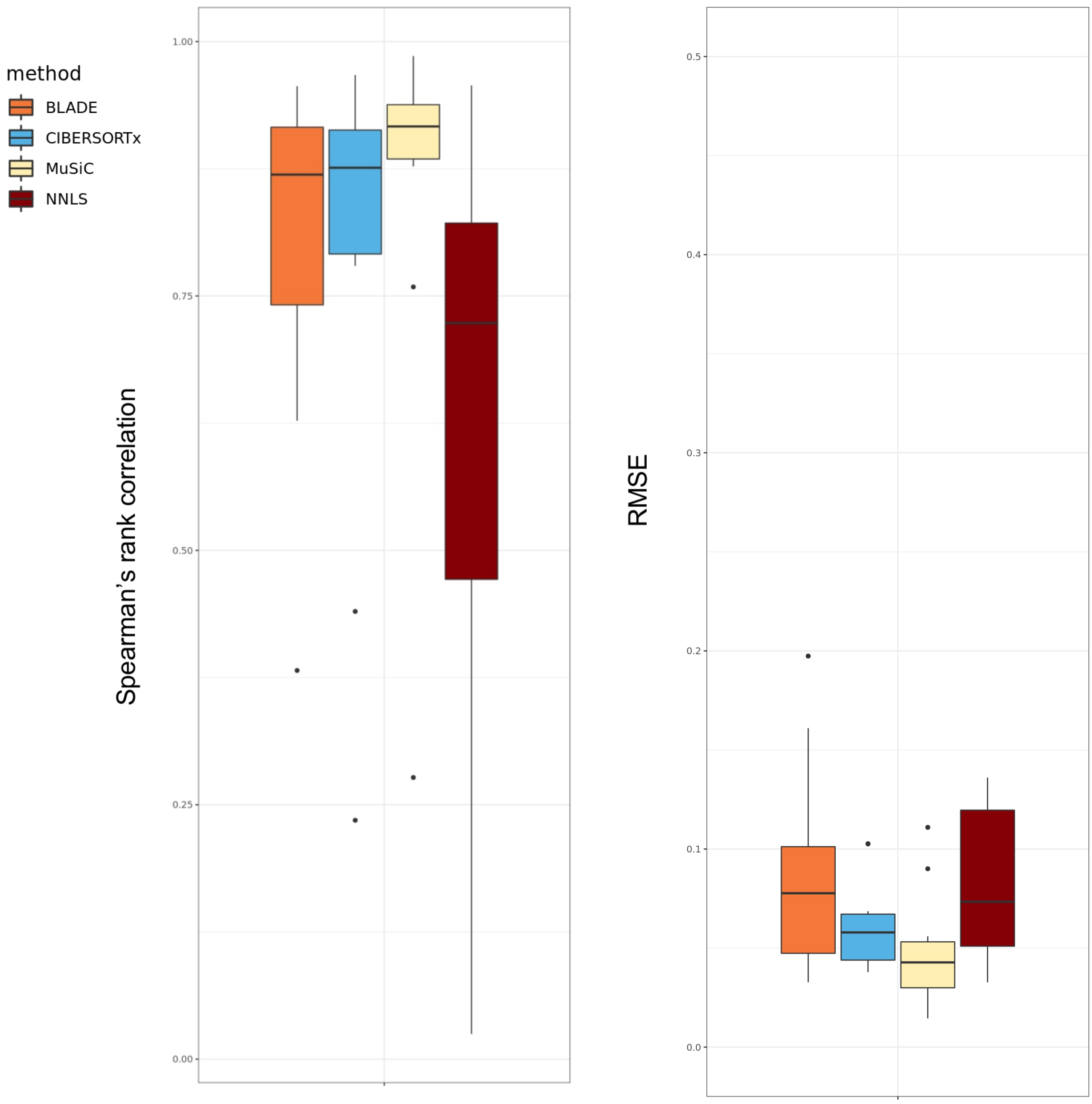

Supplementary Figure S24. Alternative performance metrics for deconvolution of PDAC RNA-seq data. Performances (Spearman's rank correlation coefficient (left) and RMSE (right); y-axis) of BLADE (orange), CIBERSORTx (blue), MuSiC (light yellow), and NNLS (dark red) in predicting cellular fractions of the PDAC bulk RNA-seq data. The standard boxplot notation was used (lower/upper hinges - first/third quartiles; whiskers extend from the top/bottom hinges to the largest/lowest values no further than 1.5 * inter-quartile ranges).

Supplmenetary Note 1: Generic deconvolution method with known cellular compositions

1.1 Formulation and Notation

We denote:

- $i = 1, \dots, n$: index for sample i.
- $j = 1, \dots, p$: index for gene
- $t = 1, \dots, T$: index for cell type.
- y_{ij}: Bulk RNA expression for individual i and gene j
- x_{ij}^t: latent gene expression for individual i, gene j and cell type t
- f_i^t: given fraction of cells of type t.

And thus y_{ij} is a (weighted) convolution:

$$y_{ij} = \sum_{t=1}^{T} f_i^t x_{ij}^t, \tag{1}$$

which implies, with $\hat{x}_{ij}^t = f_i^t x_{ij}^t$,

$$g_{y_{ij}}(y) = \int_{u_1=0}^{y} \cdots \int_{u_T=0}^{y-\sum_{i=1}^{T-1} u_i} g_{\hat{x}_{ij}^1}(u_1) \cdots g_{\hat{x}_{ij}^{T-1}}(u_{T-1}) g_{\hat{x}_{ij}^T}(y - \sum_{i=1}^{T-1} u_i) du_1 \dots du_T. \tag{2}$$

If we assume $x_{ij}^t \sim LN(\mu_j^t, (\sigma_j^t)^2)$ then $\hat{x}_{ij}^t = f_i^t x_{ij}^t \sim LN(\log(f_i^t) + \mu_j^t, (\sigma j^t)^2)$. Therefore, y_{ij} is a convolution of T log-normal random variables.

1.2 Approximation of Log-Normal (LN)/Negative Binomial (NB) convolution using Probabilistic Generative Function (PGF)

Note that evaluation of $g_{y_{ij}}(y)$ needs to be extremely efficient, because for numerical maximum likelihood estimation $g_{y_{ij}}(y)$ is evaluated many times, depending on the number of parameters (I times J times T). While numerical evaluation of (2) may still be efficient for $T = 2$[1], the extension to $T > 2$ is not straightforward to a $T - 1$ dimensional integral. To this end, the log-normal density $g_t = g_{\hat{x}_{ij}^t}$ is approximated by a probability generating function (PGF).

Specifically, we used an equi-spaced grid that ranges from 0 to $y = y_{ij}$ (i.e., observed convoluted gene expression level of sample i and gene j) in which the bins represent integer fractions of y, centered around those fractions. For instance, for $y = 100$, there are 101 bins representing $0, \dots, 100$, with bin $i : [i - 0.5, i + 0.5]$. For each bin $i : [a_i, a_{i+1}]$ a total density of $p_i = G_t(a_{i+1}) - G_t(a_i)$ is assigned, where G_t is the cumulative distribution of $\hat{x}_i j^t$. Then, the PGF of the associated approximation density $\hat{g}_t$ is:

$$G_{\hat{g}_t}(x) = \sum_{k=0}^{B} p_k x^k \tag{3}$$

By assuming that y is an integer and the bins represent $0, \dots, y$ we have

$$G_{\hat{g}}(x) = \prod_{t=1}^{T} G_{\hat{g}_t}(x) \tag{4}$$

Hence,

$$g_{y_{ij}}(y) = G_{\hat{g}}(x)[x^y], \tag{5}$$

which is simply the coefficient of x^y in the product (4). Thanks to the equi-spaced grid, $G_{\hat{g}_t}(x)$ is a polynomial, for which repeated multiplication is very efficient. In fact, since there is x^y term at the end (because this represents observation y), a higher order can be discarded for the multiplications in (4). Therefore, when using (4) computation grows only linearly with T, as opposed to (naive) evaluation of (2). The R package *pracma* was used for efficient polynomial multiplication.

For discrete distributions such as the negative binomial, (4) is exact when the number of bins equals the total count plus one. PGF requires to evaluate probabilities for bins $[a_i, a_{i+1}]$. Cumulative distribution G_t is calculated as follows:

$$G_t(x) = P(\hat{x}_{ij}^t \leq x) = P(x_{ij}^t f_i^t \leq x) = P(x_{ij}^t \leq x/f_i^t) = G^{NB}(x/f_i^t) \quad (6)$$

where G^{NB} denotes the cumulative distribution function of the negative binomial with parameters (μ_j^t, σ_j^t). With given fractions f_i^t and convolution $g_{y_{ij}}$, the log-likelihood for gene j equals:

$$\mathcal{L}(Y_{.j}; \theta_j) = \sum_{i=1}^{I} \log g_{y_{ij}}(y_{ij}; \theta_j) \quad (7)$$

where θ_j contains all $2T$ parameters of the convolution components and evaluated by (5). The MLE estimate of θ_j is obtained by maximizing $\mathcal{L}(Y_{.j}; \theta_j)$ using the numerical optimizer *Rsolnp*. The numerical optimizer requires several thousands of evaluations of (2) per gene, which limits the scalability of the method.

1.3 Evaluation of the PGF approximation of LN convolution model

Unlike NB distribution for which PGF approximation is exact, LN distribution is a continuous distribution and thus PGF is not exact. Therefore, we evaluated the accuracy of PGF approximation for LN, taking an alternative approximation method, Fenton-Wilkinson (FW) approximation[2], for a comparison. FW approximates $g_y = g_{y_{ij}}(y)$ by a log-normal density, thanks to which FW is free of numerical integration and thus computationally very efficient. As a reference, we also implemented Monte-Carlo (MC) evaluation of (2), which is computationally intense ($M = 10^6$ sampling) but accurate. Afterwards, the likelihood of PGF and FW approximation were evaluated based on the a simulation data with three cell types ($T = 3$) using the following steps:

- Means and standard deviations of 3 cell types are sampled from the uniform distribution: $\mu^t \sim U[0, 3]$, $\sigma^t \sim U[0.5, 2]$.
- Simulation bulk gene expression data is sampled as follows: $Y = Z^1 + Z^2 + Z^3$, where $Z^t \sim LN(\mu^t, (\sigma^t)^2)$.
- Evaluate the likelihood for 3 realizations of $Y = y$: $y = \mu_Y/2$, μ_Y, and $2\mu_Y$, where μ_Y is the expectation of Y: $\mu_Y = \exp(\mu^1 + (\sigma^1)^2/2) + \exp(\mu^2 + (\sigma^2)^2/2) + \exp(\mu^3 + (\sigma^3)^2/2)$.

The approximated likelihoods by PGF and FW are compared to the reference likelihood calculated by MC, in which we found a superior concordance of PGF with MC (see **Supplementary Figure S3**).

Supplmenetary Note 2: Detailed derivation of BLADE

In this section, we will provide a detailed derivation of Bayesian-lognormal deconvolution with a collapsed variational inference.

2.1 Notation

We assume there are expression levels of J genes obtaind from I samples. The bulk gene expression data is contributed by T cell types. We will define the following variables:

- y_{ij}: log-transformed bulk expression levels of gene j for sample i.
- x_{ij}^t: log-transformed expression level of gene j in cell type t in sample i.
- f_i^t: Fraction of cell type t in sample i. (where $\sum_t f_i^t = 1$ and $f_i^t \geq 0$),

where y_{ij} is observed variable, while x_{ij}^t and f_i^t are hidden variables. Then, we will assume the following deconvolution problem.

$$y_{ij} = \log\left(\sum_t f_i^t \exp(x_{ij}^t)\right) + \epsilon \tag{8}$$

Note that we assume y_{ij} and x_{ij}^t to be log-transformed data for the convenience to use Gaussian distribution instead of the log-normal distribution. In the linear scale, $\exp(y_{ij})$ and $\exp(x_{ij}^t)$, these variables follows log-normal distribution and actual deconvolution is done on linear-scale.

2.2 Probabilistic assumptions

For the three random variables, we assume the following underlining probability distributions.

$$\begin{aligned}
P(x_{ij}^t|\mu_j^t, \lambda_j^t) &= \mathcal{N}\left(x_{ij}^t|\mu_j^t, \frac{1}{\lambda_j^t}\right) && (9)\\
P(f_i^1, ..., f_i^T|\alpha_i^1, ..., \alpha_i^T) &= \mathcal{D}\left(f_i^1, ..., f_i^T|\alpha_i^1, ..., \alpha_i^T\right) && (10)\\
P(y_{ij}|x_{ij}^t, f_i^1, ..., f_i^T, \lambda_{ij}) &= \mathcal{N}\left(y_{ij}|\log\left(\sum_t \exp(x_{ij}^t) f_i^t\right), \frac{1}{\lambda_{ij}}\right) && (11)
\end{aligned}$$

Note that λ in $\mathcal{N}(x|\mu, \frac{1}{\lambda})$ is a precision, inverse of which is the variance. To incorporate the prior knowledge of gene expression profiles per cell type, we take a Bayesian framework for the x_{ij}^t, endowing conjugate prior distribution for the parameters, μ_j^t and λ_j^t, as the follows:

$$P(\mu_j^t, \lambda_j^t|\mu_{0j}^t, \kappa_{0j}^t, \alpha_{0j}^t, \beta_{0j}^t) = \mathcal{N}\left(\mu_j^t|\mu_{0j}^t, \frac{1}{\kappa_{0j}^t \lambda_j^t}\right) \mathcal{G}(\lambda_j^t|\alpha_{0j}^t, \beta_{0j}^t) \tag{12}$$

The hyperparameters are chosen based on the observed expression levels and standard deviation of each gene and each cell type, derived from the single-cell RNA-seq data (see Online Methods).

2.3 Optimization - collapsed variational inference

We take a collapsed variational inference for the optimization of the model [3]. Denote the hidden variables in our model by Z, and the observed one as Y. The standard approach is to maximize the evidence lower-bound (ELBO):

$$\log P(Y) \geq \int Q(Z) \log\left(\frac{P(Y, Z|\theta)}{Q(Z)}\right) dZ \tag{13}$$

where $Q(Z)$ is the variational distribution which approximates the intractable posterior probability of hidden variables given observed variables (i.e., $P(Z|Y)$). Then maximization of 13 w.r.t. the variational parameters of $Q(Z)$ is equivalent to minimization of the Kullback-Leibler divergence between $P(Z|Y)$ and $Q(Z)$ [4], which should render $Q(Z)$ to be an accurate approximation of $P(Z|Y)$. Before discussion the specific variational distributions for X (all latent gene expressions) and F (all latent cell type fractions), a subset of hidden variables are integrated out in advance to reduce the complexity.

2.3.1 Collapsing conjugate priors

We first integrate out the variables with conjugate prior distribution (i.e., collapsing the variables). This allows us to account for the entire distribution of the variables in a fully Bayesian manner, instead of finding maximum a posteriori solution. In our model, μ_j^t and λ_j^t endowed with a conjugate prior, which can be integrated out as follows:

$$\prod_{\forall_i} P(x_{ij}^t|\mu_{0j}^t,\kappa_{0j}^t,\alpha_{0j}^t,\beta_{0j}^t) = \int \prod_{\forall_i} P(x_{ij}^t|\mu_j^t,\lambda_j^t)P(\mu_j^t,\lambda_j^t|\mu_{0j}^t,\kappa_{0j}^t,\alpha_{0j}^t,\beta_{0j}^t)d\mu_j^t d\lambda_j^t$$

$$= \frac{\Gamma(\alpha_{nj}^t){\beta_{0j}^t}^{\alpha_{0j}^t}}{\Gamma(\alpha_{0j}^t){\beta_{nj}^t}^{\alpha_{nj}^t}}\left(\frac{\kappa_{0j}^t}{\kappa_{nj}^t}\right)^{\frac{1}{2}}(2\pi)^{-1/2} \quad (14)$$

where

- $\alpha_{nj}^t = \alpha_{0j}^t + \frac{I}{2}$
- $\kappa_{nj}^t = \kappa_{0j}^t + I$
- $\beta_{nj}^t = \beta_{0j}^t + \frac{1}{2}\sum_{i=1}^I (x_{ij}^t - \hat{x_j^t})^2 + \frac{\kappa_{0j}^t I(\hat{x_j^t}-\mu_{0j}^t)^2}{2(\kappa_{0j}^t+I)}$
- $\hat{x_j^t} = \frac{1}{I}\sum_{i=1}^I x_{ij}^t$

By replacing $P(x_{ij}^t|\mu_j^t,\lambda_j^t)P(\mu_j^t,\lambda_j^t|\mu_{0j}^t,\kappa_{0j}^t,\alpha_{0j}^t,\beta_{0j}^t)$ with the marginal distribution $P(x_{ij}^t|\mu_{0j}^t,\kappa_{0j}^t,\alpha_{0j}^t,\beta_{0j}^t)$, we eliminate the parameters μ_j^t and λ_j^t from the model.

2.3.2 Variational distribution

After collapsing μ_j^t and λ_j^t, we assume the following variational distributions for the remaining hidden variables:

$$Q(x_{ij}^t|\nu_{ij}^t,\omega_j^t) = \mathcal{N}(x_{ij}^t|\nu_{ij}^t,\omega_j^t) \quad (15)$$

$$Q(f_i^1,...,f_i^T|\beta_i^1,...,\beta_i^T) = \mathcal{D}\left(f_i^1,...,f_i^T|\beta_i^1,...,\beta_i^T\right) \quad (16)$$

ν_{ij}^t,ω_j^t and β_i^t ($\forall t = 1,\ldots,T;\ \forall i = 1,\ldots,I;\ \forall j = 1,\ldots,J$) are the variational parameters to be optimized in our model.

2.4 Optimization of variational parameters: Evidence lower-bound (ELBO)

Given the variational distribution $Q(\cdot)$, the ELBO 13 is optimized with respect to the variational parameters. To achieve this, we take a line-search approach using Limited-memory Broyden-Fletcher-Goldfarb-Shannon (L-BFGS) algorithm. For efficient optimization, both the objective function and gradients of the parameters to be optimized are analytically calculated.

The ELBO 13 can be analytically calculated as follows:

$$\int Q(X,F)\log\left(\frac{P(Y,X,F|\theta)}{Q(X,F)}\right)dZ = \int Q(X)Q(F)\log\left(\frac{P(Y|X,F)P(X)P(F)}{Q(X)Q(F)}\right)dZ$$
$$= \underbrace{\mathbb{E}_Q[\log(P(X|\mu_0,\kappa_0,\alpha_0,\beta_0))]}_{1} + \underbrace{\mathbb{E}_Q[\log(P(Y|X,F,\sigma)]}_{2} + \underbrace{\mathbb{E}_Q[\log(P(F|\alpha))]}_{3}$$
$$- \underbrace{\mathbb{E}_Q[\log(Q(X|\nu,\omega))]}_{4} - \underbrace{\mathbb{E}_Q[\log(Q(F|\beta))]}_{5} \quad (17)$$

See appendix for the calculation of the five components in (17) and its derivaties with respect to the variational parameters.

2.5 Empirical Bayes approach for selection of hyperparameters

BLADE has the following hyperparameters, among which a subset is determined automatically.

- f_i^t: $P(f_i^1, \dots, f_i^T | \alpha_i^1, \dots, \alpha_i^T)$
  - α_i^t: Parameters for Dirichlet distribution ($\alpha_i^t \geq 0$).
- x_{ij}^t: $P(x_{ij}^t | \mu_{0j}^t, \kappa_{0j}^t, \alpha_{0j}^t, \beta_{0j}^t)$
  - μ_{0j}^t: Expected expression level of gene j in cell type t in log-scale (i.e., $\mathbb{E}[x_j^t]$). This is estimated using scRNA-seq data.
  - κ_{0j}^t: A scaling factgor for precision of μ_j^t.
  - α_{0j}^t: A shape parameter for Gamma distribution.
  - β_{0j}^t: A rate paramter, which we set $\beta_{0j}^t = \alpha_{0j}^t \mathbb{V}[x_j^t]$, where $\mathbb{V}[x_j^t]$ is the sample variance of gene j in cell type t measured in scRNA-seq data. This is based on the fact that the precision of gene j (i.e., inverse of variance; $\lambda_j^t = \frac{1}{\mathbb{V}[x_j^t]}$) follows $\mathcal{G}(\alpha_{0j}^t, \beta_{0j}^t)$ and its expectation is $\frac{\alpha_{0j}^t}{\beta_{0j}^t}$ (i.e., $\frac{1}{\mathbb{V}[x_j^t]} = \frac{\alpha_{0j}^t}{\beta_{0j}^t}$). This allows us to incorporate gene expression variability observed in scRNA-seq data.
- y_{ij}: $P\left(y_{ij} | \log\left(\sum_t f_i^t \exp(x_{ij}^t)\right), \frac{1}{\tau_j}\right)$
  - τ_j: Precision per gene. We chose $\tau_j = \frac{1}{s\mathbb{V}[y_j]}$, where s is the user-defined scaling facor and $\mathbb{V}[y_j]$ is observed variance (i.e., inverse of precision) for gene j.

Among the hyperparameters, α_i^t, κ_{0j}^t, α_{0j}^t, and s is chosen by the users. To systematically identify the optimial parameters, we employed an Empirical Bayes approach to select the best set of parameters. For each configuration of parameters, we obtained maximum likelihood estimates using only a subset of samples. We only used a subset of samples, not only to gain computational efficiency but also to avoid overfitting. Then, the hyper-parameter configuration with the highest likelihood, maximized w.r.t. the model parameters, is selected, followed by performing deconvolution using the entire data set. Throughout the manuscript, we considered a total of 90 different parameter configurations that cover all possible combinations of: $\alpha_i^t \in \{1, 10\}$, $\alpha_{0j}^t \in \{0.1, 0.5, 1, 5, 10\}$, $\kappa_{0j}^t \in \{1, 0.5, 0.1\}$, and $s \in \{1, 0.3, 0.5\}$.

References

[1] Ahn, J. *et al.* DeMix: deconvolution for mixed cancer transcriptomes using raw measured data. *Bioinformatics* **29**, 1865–1871 (2013).

[2] Fenton, L. The sum of log-normal probability distributions in scatter transmission systems. *IRE Transactions on Communications Systems* **8**, 57–67 (1960).

[3] Teh, Y. W., Kurihara, K. & Welling, M. Collapsed variational inference for hdp. In Platt, J. C., Koller, D., Singer, Y. & Roweis, S. T. (eds.) *Advances in Neural Information Processing Systems 20*, 1481–1488 (Curran Associates, Inc., 2008). URL `http://papers.nips.cc/paper/3342-collapsed-variational-inference-for-hdp.pdf`.

[4] Blei, D. M., Kucukelbir, A. & McAuliffe, J. D. Variational inference: A review for statisticians. *Journal of the American Statistical Association* **112**, 859?877 (2017). URL `http://dx.doi.org/10.1080/01621459.2017.1285773`.

Appendix

A.1 Derivation of the ELBO function

Each of the five components in (17) can be analytically calculated as below:

$$1 : \mathbb{E}_Q[\log(P(X|\mu_0, \kappa_0, \alpha_0, \beta_0))]$$
$$= -\sum_{\forall j,t} \alpha^t_{nj} \mathbb{E}_Q \left[\log \left(\beta^t_{0j} + \frac{1}{2} \sum_{i=1}^{I} (x^t_{ij} - \hat{x^t_j})^2 + \frac{\kappa^t_{0j} I (\hat{x^t_j} - \mu^t_{0j})^2}{2(\kappa^t_{0j} + I)} \right) \right] + const$$
$$\geq -\sum_{\forall j,t} \alpha^t_{nj} \log \left(\beta^t_{0j} + \frac{(I-1)}{2} (\omega^t_j)^2 + \frac{1}{2} \sum_{i=1}^{I} \left(\nu^t_{ij} - \hat{\nu^t_j} \right)^2 + \frac{\kappa^t_{0j} I}{2(\kappa^t_{0j} + I)} \left(\frac{1}{I} (\omega^t_j)^2 + (\hat{\nu^t_j} - \mu^t_{0j})^2 \right) \right) + const \tag{18}$$

where

$$\hat{\nu^t_j} = \frac{1}{I} \sum_i \nu^t_{ij} \tag{19}$$

The inequality is introduced by Jensen's inequality, by making use of the convex property of the function $f(x) = -\log(x)$.

$$2 : \mathbb{E}_Q[\log(P(Y|X, F, \sigma)] = \sum_{\forall (i,j)} \left(-\frac{1}{2\sigma^2_{ij}} \left(\underbrace{\mathbb{V}_Q[\log(\sum_t \exp(x^t_{ij}) f^t_i)]}_{a} + \underbrace{\mathbb{E}_Q[y_{ij} - \log(\sum_t \exp(x^t_{ij}) f^t_i)]^2}_{b} \right) \right), \tag{20}$$

$$a : \mathbb{V}_Q[\log(\sum_t \exp(x^t_{ij}) f^t_i)]$$
$$\approx \left(\frac{1}{\mathbb{E}_Q[\sum_t \exp(x^t_{ij}) f^t_i]} \right)^2 \mathbb{V}_Q \left[\sum_t \exp(x^t_{ij}) f^t_i \right] = \frac{\mathbb{V}_Q \left[\sum_t \exp(x^t_{ij}) f^t_i \right]}{\mathbb{E}_Q[\sum_t \exp(x^t_{ij}) f^t_i]^2}, \tag{21}$$

$$b : \mathbb{E}_Q[y_{ij} - \log(\sum_t \exp(x^t_{ij}) f^t_i)]^2$$
$$\approx \left(\left(y_{ij} - \log(\mathbb{E}_Q[\sum_t \exp(x^t_{ij}) f^t_i)]) \right) - \frac{\mathbb{V}_Q[\sum_t \exp(x^t_{ij}) f^t_i)]}{2\mathbb{E}_Q[\sum_t \exp(x^t_{ij}) f^t_i)]^2} \right)^2 \tag{22}$$

which is based on the laplace apporixmation: $f(x) \approx f(x_0) - \frac{1}{2}|f''(x_0)|(x - x_0)^2$. The expectation and variance terms can be calculated as below:

$$\mathbb{E}_Q \left[\sum_t \exp(x^t_{ij}) f^t_i \right] = \sum_t \exp \left(\nu^t_{ij} + \frac{(\omega^t_j)^2}{2} \right) \frac{\beta^t_i}{\sum_k \beta^k_i} \tag{23}$$

$$\mathbb{V}_Q\left[\sum_t \exp(x_{ij}^t)f_i^t\right] = \sum_t \mathbb{V}_Q[\exp(x_{ij}^t)f_i^t] + \sum_{\forall(l,k)l\neq k} \mathbb{E}_Q[\exp(x_{ij}^l)\exp(x_{ij}^k)]\mathbb{C}_Q[f_i^l, f_i^k]$$

$$= \sum_t \left(\exp\left(2\nu_{ij}^t + 2(\omega_j^t)^2\right)\left(\frac{\tilde{\beta}_i^t(1-\tilde{\beta}_i^t)}{\beta_{i0}+1} + \tilde{\beta}_i^{t\,2}\right) - \exp\left(2\nu_{ij}^t + (\omega_j^t)^2\right)\tilde{\beta}_i^{t\,2}\right)$$

$$+ \sum_{\forall(l,k)l\neq k} \exp\left(\nu_{ij}^k + \nu_{ij}^l + \frac{(\omega_j^k)^2 + (\omega_j^l)^2}{2}\right)\frac{-\tilde{\beta}_i^k\tilde{\beta}_i^l}{\beta_{i0}+1} \quad (24)$$

$$3 \;:\; \mathbb{E}_Q[\log(P(F|\alpha))] \;=\; \sum_i \left(-\log\left(\frac{\prod_t \Gamma(\alpha_i^t)}{\Gamma(\sum_t \alpha_i^t)}\right) + \sum_t (\alpha_i^t - 1)\left(\phi(\beta_i^t) - \phi(\sum_t \beta_i^t)\right)\right) \quad (25)$$

$$4 : \mathbb{E}_Q[\log(Q(X|\nu,\omega))] = -\frac{1}{2}\sum_{\forall(i,j,t)}\left(\log(2\pi(\omega_j^t)^2) - 1\right) \quad (26)$$

$$5 \;:\; \mathbb{E}_Q[\log(Q(F|\beta))] \;=\; \sum_i \left(-\log B(\beta_i) + (\beta_i^t - 1)\left(\phi(\beta_i^t) - \phi(\sum_t \beta_i^t)\right)\right) \quad (27)$$

A.2 Gradient of ν_{ij}^t

The gradient of ν_{ij}^t is calculated for each of the 5 components in 17.

$$\frac{\partial}{\partial \nu_{ij}^t}1$$

$$= \frac{\partial}{\partial \nu_{ij}^t}\left(-\sum_{\forall j,t}\alpha_{nj}^t \log\left(\beta_{0j}^t + \frac{I(I+3)}{2}(\omega_j^t)^2 + \frac{1}{2}\sum_{i=1}^I\left(\nu_{ij}^t - \hat{\nu_j^t}\right)^2 + \frac{\kappa_{0j}^t I}{2(\kappa_{0j}^t + I)}\left(\frac{1}{I}(\omega_j^t)^2 + (\hat{\nu_j^t} - \mu_{0j}^t)^2\right)\right)\right)$$

$$= -\alpha_{nj}^t \frac{\nu_{ij}^t - \hat{\nu_j^t} - \frac{1}{T}\sum_k(\nu_{kj}^t - \hat{\nu_j^t}) + \frac{\kappa_{0j}^t}{(\kappa_{0j}^t+I)}\left(\hat{\nu_j^t} - \mu_{0j}^t\right)}{\left(\beta_{0j}^t + \frac{(I-1)}{2}(\omega_j^t)^2 + \frac{1}{2}\sum_{i=1}^I\left(\nu_{ij}^t - \hat{\nu_j^t}\right)^2 + \frac{\kappa_{0j}^t I}{2(\kappa_{0j}^t+I)}\left(\frac{1}{I}(\omega_j^t)^2 + (\hat{\nu_j^t} - \mu_{0j}^t)^2\right)\right)} \quad (28)$$

$$\frac{\partial}{\partial \nu_{ij}^t}2 =$$

$$-\frac{1}{2\sigma_{ij}^2}\left(\underbrace{\frac{\partial}{\partial \nu_{ij}^t}\frac{\mathbb{V}_Q\left[\sum_t \exp(x_{ij}^t)f_i^t\right]}{\mathbb{E}_Q[\sum_t \exp(x_{ij}^t)f_i^t]^2}}_{a} + \underbrace{\frac{\partial}{\partial \nu_{ij}^t}\left(\left(y_{ij} - \log(\mathbb{E}_Q[\sum_t \exp(x_{ij}^t)f_i^t])\right) - \frac{\mathbb{V}_Q[\sum_t \exp(x_{ij}^t)f_i^t)]}{2\mathbb{E}_Q[\sum_t \exp(x_{ij}^t)f_i^t)]^2}\right)^2}_{b}\right) \quad (29)$$

$$a : \frac{\partial}{\partial \nu_{ij}^t}\frac{\mathbb{V}_Q\left[\sum_t \exp(x_{ij}^t)f_i^t\right]}{\mathbb{E}_Q[\sum_t \exp(x_{ij}^t)f_i^t]^2} = \frac{\partial(\mathbb{V}_Q[\cdot])\mathbb{E}_Q[\cdot] - 2\partial(\mathbb{E}_Q[\cdot])\mathbb{V}_Q[\cdot]}{\mathbb{E}_Q[\cdot]^3} \quad (30)$$

$$b : \frac{\partial}{\partial \nu_{ij}^t}\left(\left(y_{ij} - \log(\mathbb{E}_Q[\sum_t \exp(x_{ij}^t) f_i^t])\right) - \frac{\mathbb{V}_Q[\sum_t \exp(x_{ij}^t) f_i^t)]}{2\mathbb{E}_Q[\sum_t \exp(x_{ij}^t) f_i^t)]^2}\right)^2$$

$$= -\left((y_{ij} - \log(\mathbb{E}_Q[\cdot])) - \frac{\mathbb{V}_Q[\cdot]}{2\mathbb{E}_Q[\cdot]^2}\right)\left(2\frac{\partial(\mathbb{E}_Q[\cdot])}{\mathbb{E}_Q[\cdot]} + \underbrace{\partial\left(\frac{\mathbb{V}_Q[\cdot]}{\mathbb{E}_Q[\cdot]^2}\right)}_{a}\right) \quad (31)$$

$$\partial(\mathbb{E}_Q[\cdot]) \quad = \quad \frac{\partial}{\partial \nu_{ij}^t}\left(\sum_t \exp\left(\nu_{ij}^t + \frac{(\omega_j^t)^2}{2}\right)\frac{\beta_i^t}{\sum_k \beta_i^k}\right) \quad = \quad \exp\left(\nu_{ij}^t + \frac{(\omega_j^t)^2}{2}\right)\frac{\beta_i^t}{\sum_k \beta_i^k} \quad (32)$$

$$\partial(\mathbb{V}_Q[\cdot]) = \frac{\partial}{\partial \nu_{ij}^t}\left(\sum_t \left(\exp\left(2\nu_{ij}^t + 2(\omega_j^t)^2\right)\left(\frac{\tilde{\beta}_i^t(1-\tilde{\beta}_i^t)}{\beta_{i0}+1} + \tilde{\beta_i^t}^2\right) - \exp\left(2\nu_{ij}^t + (\omega_j^t)^2\right)\tilde{\beta_i^t}^2\right)\right.$$
$$\left. + \sum_{\forall (l,k) l \neq k} \exp\left(\nu_{ij}^k + \nu_{ij}^l + \frac{(\omega_j^k)^2 + (\omega_j^l)^2}{2}\right)\frac{-\tilde{\beta}_i^k \tilde{\beta}_i^l}{\beta_{i0}+1}\right)$$
$$= 2\exp\left(2\nu_{ij}^t + 2(\omega_j^t)^2\right)\left(\frac{\tilde{\beta}_i^t(1-\tilde{\beta}_i^t)}{\beta_{i0}+1} + \tilde{\beta_i^t}^2\right) - 2\exp\left(2\nu_{ij}^t + (\omega_j^t)^2\right)\tilde{\beta_i^t}^2$$
$$+ \sum_{k \neq t} \exp\left(\nu_{ij}^t + \nu_{ij}^k + \frac{(\omega_j^t)^2 + (\omega_k^l)^2}{2}\right)\frac{-\tilde{\beta}_i^t \tilde{\beta}_i^k}{\beta_{i0}+1} \quad (33)$$

The other components in 17 does not involve ν_{ij}^t, therefore

$$\frac{\partial}{\partial \nu_{ij}^t} 3 = \frac{\partial}{\partial \nu_{ij}^t} 4 = \frac{\partial}{\partial \nu_{ij}^t} 5 = 0. \quad (34)$$

A.3 Gradient of ω_j^t

$$\frac{\partial}{\partial \omega_j^t} 1 =$$
$$\frac{\partial}{\partial \omega_j^t}\left(-\sum_{\forall j,t} \alpha_{nj}^t \log\left(\beta_{0j}^t + \frac{I(I+3)}{2}(\omega_j^t)^2 + \frac{1}{2}\sum_{i=1}^{I}\left(\nu_{ij}^t - \hat{\nu_j^t}\right)^2 + \frac{\kappa_{0j}^t I}{2(\kappa_{0j}^t + I)}\left(\frac{1}{I}(\omega_j^t)^2 + (\hat{\nu_j^t} - \mu_{0j}^t)^2\right)\right)\right)$$
$$= -\alpha_{nj}^t \frac{(I-1)\omega_j^t + \frac{\kappa_{0j}^t}{(\kappa_{0j}^t + I)}\omega_j^t}{\left(\beta_{0j}^t + \frac{(I-1)}{2}(\omega_j^t)^2 + \frac{1}{2}\sum_{i=1}^{I}\left(\nu_{ij}^t - \hat{\nu_j^t}\right)^2 + \frac{\kappa_{0j}^t I}{2(\kappa_{0j}^t + I)}\left(\frac{1}{I}(\omega_j^t)^2 + (\hat{\nu_j^t} - \mu_{0j}^t)^2\right)\right)} \quad (35)$$

$$\frac{\partial}{\partial \omega_j^t} 2 = -\sum_{\forall i} \frac{1}{2\sigma_{ij}^2}\left(\underbrace{\frac{\partial}{\partial \omega_j^t}\frac{\mathbb{V}_Q\left[\sum_t \exp(x_{ij}^t) f_i^t\right]}{\mathbb{E}_Q[\sum_t \exp(x_{ij}^t) f_i^t]^2}}_{a} + \right.$$
$$\left.\underbrace{\frac{\partial}{\partial \omega_j^t}\left(\left(y_{ij} - \log(\mathbb{E}_Q[\sum_t \exp(x_{ij}^t) f_i^t)])\right) - \frac{\mathbb{V}_Q[\sum_t \exp(x_{ij}^t) f_i^t)]}{2\mathbb{E}_Q[\sum_t \exp(x_{ij}^t) f_i^t)]^2}\right)^2}_{b}\right) \quad (36)$$

$$a : \frac{\partial}{\partial \omega_j^t} \frac{\mathbb{V}_Q\left[\sum_t \exp(x_{ij}^t) f_i^t\right]}{\mathbb{E}_Q[\sum_t \exp(x_{ij}^t) f_i^t]^2} = \frac{\partial(\mathbb{V}_Q[\cdot])\mathbb{E}_Q[\cdot] - 2\partial(\mathbb{E}_Q[\cdot])\mathbb{V}_Q[\cdot]}{\mathbb{E}_Q[\cdot]^3} \tag{37}$$

$$\begin{aligned} b : \frac{\partial}{\partial \omega_j^t} & \left(\left(y_{ij} - \log(\mathbb{E}_Q[\sum_t \exp(x_{ij}^t) f_i^t)]) \right) - \frac{\mathbb{V}_Q[\sum_t \exp(x_{ij}^t) f_i^t)]}{2\mathbb{E}_Q[\sum_t \exp(x_{ij}^t) f_i^t)]^2} \right)^2 \\ &= -\left((y_{ij} - \log(\mathbb{E}_Q[\cdot])) - \frac{\mathbb{V}_Q[\cdot]}{2\mathbb{E}_Q[\cdot]^2} \right) \left(2\frac{\partial(\mathbb{E}_Q[\cdot])}{\mathbb{E}_Q[\cdot]} + \underbrace{\partial\left(\frac{\mathbb{V}_Q[\cdot]}{\mathbb{E}_Q[\cdot]^2}\right)}_{a} \right) \end{aligned} \tag{38}$$

$$\partial(\mathbb{E}_Q[\cdot]) \quad = \quad \frac{\partial}{\partial \omega_j^t} \left(\sum_t \exp\left(\nu_{ij}^t + \frac{(\omega_j^t)^2}{2} \right) \frac{\beta_i^t}{\sum_k \beta_i^k} \right) \quad = \quad \exp\left(\nu_{ij}^t + \frac{(\omega_j^t)^2}{2} \right) \frac{\beta_i^t}{\sum_k \beta_i^k} \omega_j^t \tag{39}$$

$$\begin{aligned} \partial(\mathbb{V}_Q[\cdot]) = \frac{\partial}{\partial \omega_j^t} & \left(\sum_t \left(\exp\left(2\nu_{ij}^t + 2(\omega_j^t)^2\right) \left(\frac{\tilde{\beta}_i^t(1-\tilde{\beta}_i^t)}{\beta_{i0}+1} + \tilde{\beta}_i^{t\,2} \right) - \exp\left(2\nu_{ij}^t + (\omega_j^t)^2\right) \tilde{\beta}_i^{t\,2} \right) \right. \\ & \left. + \sum_{\forall (l,k) l \neq k} \exp\left(\nu_{ij}^k + \nu_{ij}^l + \frac{(\omega_j^k)^2 + (\omega_j^l)^2}{2} \right) \frac{-\tilde{\beta}_i^k \tilde{\beta}_i^l}{\beta_{i0}+1} \right) \\ = 4\omega_j^t & \exp\left(2\nu_{ij}^t + 2(\omega_j^t)^2\right) \left(\frac{\tilde{\beta}_i^t(1-\tilde{\beta}_i^t)}{\beta_{i0}+1} + \tilde{\beta}_i^{t\,2} \right) - 2\omega_j^t \exp\left(2\nu_{ij}^t + (\omega_j^t)^2\right) \tilde{\beta}_i^{t\,2} \\ & + \sum_{k \neq t} \exp\left(\nu_{ij}^t + \nu_{ij}^k + \frac{(\omega_j^t)^2 + (\omega_k^l)^2}{2} \right) \frac{-\tilde{\beta}_i^t \tilde{\beta}_i^k}{\beta_{i0}+1} \omega_j^t \end{aligned} \tag{40}$$

$$\frac{\partial}{\partial \omega_j^t} 4 \quad = \quad \frac{\partial}{\partial \omega_j^t} \left(-\frac{1}{2} \sum_{\forall (i,j,t)} \left(\log(2\pi(\omega_j^t)^2) + 1 \right) \right) \quad = \quad -\frac{I}{\omega_j^t} \tag{41}$$

The other components in 17 does not involve ω_j^t, therefore

$$\frac{\partial}{\partial \omega_j^t} 3 = \frac{\partial}{\partial \omega_j^t} 5 = 0. \tag{42}$$

A.4 Gradient of β_i^t

$$\begin{aligned} & \frac{\partial}{\partial \beta_i^t} 2 = \\ & -\frac{1}{2\sigma_{ij}^2} \left(\underbrace{\frac{\partial}{\partial \beta_i^t} \frac{\mathbb{V}_Q\left[\sum_t \exp(x_j^t) f_i^t\right]}{\mathbb{E}_Q[\sum_t \exp(x_j^t) f_i^t]^2}}_{a} + \underbrace{\frac{\partial}{\partial \beta_i^t} \left(\left(y_{ij} - \log(\mathbb{E}_Q[\sum_t \exp(x_j^t) f_i^t)]) \right) - \frac{\mathbb{V}_Q[\sum_t \exp(x_j^t) f_i^t)]}{2\mathbb{E}_Q[\sum_t \exp(x_j^t) f_i^t)]^2} \right)^2}_{b} \right) \end{aligned} \tag{43}$$

$$a : \frac{\partial}{\partial \beta_i^t} \frac{\mathbb{V}_Q\left[\sum_t \exp(x_j^t) f_i^t\right]}{\mathbb{E}_Q[\sum_t \exp(x_j^t) f_i^t]^2} = \frac{\partial(\mathbb{V}_Q[\cdot])\mathbb{E}_Q[\cdot] - 2\partial(\mathbb{E}_Q[\cdot])\mathbb{V}_Q[\cdot]}{\mathbb{E}_Q[\cdot]^3} \tag{44}$$

$$b:\frac{\partial}{\partial\beta_i^t}\left(\left(y_{ij}-\log(\mathbb{E}_Q[\sum_t\exp(x_j^t)f_i^t)])\right)-\frac{\mathbb{V}_Q[\sum_t\exp(x_j^t)f_i^t)]}{2\mathbb{E}_Q[\sum_t\exp(x_j^t)f_i^t)]^2}\right)^2$$

$$=-\left((y_{ij}-\log(\mathbb{E}_Q[\cdot]))-\frac{\mathbb{V}_Q[\cdot]}{2\mathbb{E}_Q[\cdot]^2}\right)\left(2\frac{\partial(\mathbb{E}_Q[\cdot])}{\mathbb{E}_Q[\cdot]}+\underbrace{\partial\left(\frac{\mathbb{V}_Q[\cdot]}{\mathbb{E}_Q[\cdot]^2}\right)}_{a}\right) \quad (45)$$

$$\partial(\mathbb{E}_Q[\cdot])=\frac{\partial}{\partial\beta_i^t}\left(\sum_t\exp\left(\nu_j^t+\frac{(\omega_j^t)^2}{2}\right)\frac{\beta_i^t}{\sum_k\beta_i^k}\right)$$

$$=\exp\left(\nu_j^t+\frac{(\omega_j^t)^2}{2}\right)\frac{1}{\sum_k\beta_i^k}-\sum_l\exp\left(\nu_j^l+\frac{(\omega_j^l)^2}{2}\right)\frac{\beta_j^l}{(\sum_k\beta_i^k)^2} \quad (46)$$

$$\partial(\mathbb{V}_Q[\cdot])=\frac{\partial}{\partial\beta_i^t}\left(\sum_t\left(\exp\left(2\nu_j^t+2(\omega_j^t)^2\right)\left(\frac{\tilde{\beta}_i^t(1-\tilde{\beta}_i^t)}{\beta_{i0}+1}+\tilde{\beta}_i^{t\,2}\right)-\exp\left(2\nu_j^t+(\omega_j^t)^2\right)\tilde{\beta}_i^{t\,2}\right)\right.$$

$$\left.+\sum_{\forall(l,k)l\neq k}\exp\left(\nu_j^k+\nu_j^l+\frac{(\omega_j^k)^2+(\omega_j^l)^2}{2}\right)\frac{-\tilde{\beta}_i^k\tilde{\beta}_i^l}{\beta_{i0}+1}\right)$$

$$=\exp\left(2\nu_j^t+2(\omega_j^t)^2\right)\left(\frac{(\beta_{i0}(\beta_{i0}+1))(\beta_{i0}-2\beta_i^t)}{(\beta_{i0})^3(\beta_{i0}+1)^2}+2\left(\frac{\beta_i^t\beta_{i0}}{\beta_{i0}^3}\right)\right)-\exp\left(2\nu_j^t+(\omega_j^t)^2\right)2\left(\frac{\beta_i^t\beta_{i0}}{\beta_{i0}^3}\right)$$

$$+\sum_k\left(\exp\left(2\nu_j^k+2(\omega_j^k)^2\right)\left(\frac{\beta_{i0}(\beta_{i0}+1)\beta_i^k-(3\beta_{i0}+2)\beta_i^k(\beta_{i0}-\beta_i^k)}{(\beta_{i0})^3(\beta_{i0}+1)^2}-2\left(\frac{(\beta_i^k)^2}{\beta_{i0}^3}\right)\right)\right.$$

$$\left.+\exp\left(2\nu_j^k+(\omega_j^k)^2\right)2\left(\frac{(\beta_i^k)^2}{\beta_{i0}^3}\right)\right)$$

$$+\sum_{\forall(l,k)l\neq k}\exp\left(\nu_j^k+\nu_j^l+\frac{(\omega_j^k)^2+(\omega_j^l)^2}{2}\right)\frac{\beta_i^k\beta_i^l(3\beta_{i0}+2)}{(\beta_{i0})^3(\beta_{i0}+1)^2}$$

$$-\sum_{\forall(k)k\neq t}\exp\left(\nu_j^t+\nu_j^k+\frac{(\omega_j^t)^2+(\omega_j^k)^2}{2}\right)\frac{\beta_i^k\beta_{i0}(\beta_{i0}+1)}{(\beta_{i0})^3(\beta_{i0}+1)^2} \quad (47)$$

$$\frac{\partial}{\partial\beta_i^t}3=\frac{\partial}{\partial\beta_i^t}\left(\sum_i\left(-\log\left(\frac{\prod_t\Gamma(\alpha_i^t)}{\Gamma(\sum_t\alpha_i^t)}\right)+\sum_t(\alpha_i^t-1)\left(\phi(\beta_i^t)-\phi(\sum_t\beta_i^t)\right)\right)\right)$$

$$=(\alpha_i^t-1)\phi'(\beta_i^t)-\sum_k(\alpha_i^k-1)\phi'\left(\sum_k\beta_i^k\right) \quad (48)$$

$$\frac{\partial}{\partial\beta_i^t}5=\frac{\partial}{\partial\beta_i^t}\left(\sum_i\left(-\log B(\beta_i)+\sum_t(\beta_i^t-1)\left(\phi(\beta_i^t)-\phi(\sum_t\beta_i^t)\right)\right)\right)$$

$$=(\beta_i^t-1)\phi'(\beta_i^t)-\sum_t(\beta_i^t-1)\phi'(\sum_t\beta_i^t) \quad (49)$$

The other components in 17 does not involve β_i^t, therefore

$$\frac{\partial}{\partial\beta_i^t}1=\frac{\partial}{\partial\beta_i^t}4=0. \quad (50)$$
