# Supplementary material for: Bayesian log-normal deconvolution for enhanced in silico microdissection of bulk gene expression data
Source: Nat Commun. 2021 Oct 20;12:6106. doi: 10.1038/s41467-021-26328-2 (PMC8528834; doi:10.1038/s41467-021-26328-2)
Supplement: Supplementary file 3 — Description of Additional Supplementary Files [file 41467_2021_26328_MOESM3_ESM.pdf]

Description of Additional Supplementary Files

File Name: Supplementary Data 1
Description: Frequencies of cell types in the 2 PBMC cite-seq data, classified in 4 different levels. Also, cell type classification for the application to real PBMC mixtures (GSE107572) is indicated.
